# Plant growth-promotion triggered by extracellular polymer is associated with facilitation of bacterial cross-feeding networks of the rhizosphere

**DOI:** 10.1093/ismejo/wraf040

**Published:** 2025-03-03

**Authors:** Yian Gu, Wenhui Yan, Yu Chen, Sijie Liu, Liang Sun, Zhe Zhang, Peng Lei, Rui Wang, Sha Li, Samiran Banerjee, Ville-Petri Friman, Hong Xu

**Affiliations:** State Key Laboratory of Materials-Oriented Chemical Engineering, Nanjing Tech University, No. 30 Puzhu South Road, Jiangbei New District, Nanjing 211816, PR China; State Key Laboratory of Materials-Oriented Chemical Engineering, Nanjing Tech University, No. 30 Puzhu South Road, Jiangbei New District, Nanjing 211816, PR China; State Key Laboratory of Materials-Oriented Chemical Engineering, Nanjing Tech University, No. 30 Puzhu South Road, Jiangbei New District, Nanjing 211816, PR China; State Key Laboratory of Materials-Oriented Chemical Engineering, Nanjing Tech University, No. 30 Puzhu South Road, Jiangbei New District, Nanjing 211816, PR China; State Key Laboratory of Materials-Oriented Chemical Engineering, Nanjing Tech University, No. 30 Puzhu South Road, Jiangbei New District, Nanjing 211816, PR China; Key Laboratory of Water-saving Agriculture of Northeast, Ministry of Agriculture and Rural Affairs, Liaoning Academy of Agricultural Science, No. 84 Dongling Road, Shenhe District, Shenyang 110161, PR China; State Key Laboratory of Materials-Oriented Chemical Engineering, Nanjing Tech University, No. 30 Puzhu South Road, Jiangbei New District, Nanjing 211816, PR China; State Key Laboratory of Materials-Oriented Chemical Engineering, Nanjing Tech University, No. 30 Puzhu South Road, Jiangbei New District, Nanjing 211816, PR China; State Key Laboratory of Materials-Oriented Chemical Engineering, Nanjing Tech University, No. 30 Puzhu South Road, Jiangbei New District, Nanjing 211816, PR China; Department of Microbiological Sciences, North Dakota State University, Van Es Hall, 1523 Centenial Blvd, Fargo, ND 58102, United States; Department of Microbiology, University of Helsinki, Viikinkaari 9, Helsinki 00014, Finland; State Key Laboratory of Materials-Oriented Chemical Engineering, Nanjing Tech University, No. 30 Puzhu South Road, Jiangbei New District, Nanjing 211816, PR China

**Keywords:** cross-feeding, poly-γ-glutamic acid, rhizosphere microbiome, plant growth-promotion

## Abstract

Despite the critical role rhizosphere microbiomes play in plant growth, manipulating microbial communities for improved plant productivity remains challenging. One reason for this is the lack of knowledge on how complex substrates secreted in the microbiome ultimately shape the microbe-microbe and plant-microbe interaction in relation to plant growth. One such complex substrate is poly-γ-glutamic acid, which is a microbially derived extracellular polymer. While it has previously been linked with plant growth-promotion, the underlying mechanisms are not well understood. Here, we show that this compound benefits plants by fostering cross-feeding networks between rhizosphere bacteria. We first experimentally demonstrate that poly-γ-glutamic acid application increases potassium bioavailability for tomato plants by driving a shift in the rhizosphere bacterial community composition. Specifically, application of poly-γ-glutamic acid increased the relative abundance of *Pseudomonas nitroreducens* L16 and *Pseudomonas monteilii* L20 bacteria which both promoted tomato potassium assimilation by secreting potassium-solubilizing pyruvic acid and potassium-chelating siderophores, respectively. Although either *Pseudomonas* strain could not metabolize poly-γ-glutamic acid directly, the application of poly-γ-glutamic acid promoted the growth of *Bacillus* species, which in turn produced metabolites that could promote the growth of both *P. nitroreducens* L16 and *P. monteilii* L20. Moreover, the *P. monteilii* L20 produced 3-hydroxycapric acid, which could promote the growth of *P. nitroreducens* L16, resulting in commensal cross-feeding interaction between plant growth-promoting bacteria. Together, these results show that poly-γ-glutamic acid plays a crucial role in driving plant growth-promotion via bacterial cross-feeding networks, highlighting the opportunity for using microbially derived, complex substrates as catalysts to increase agricultural productivity.

## Graphical Abstract

**Graphical Abstract:**
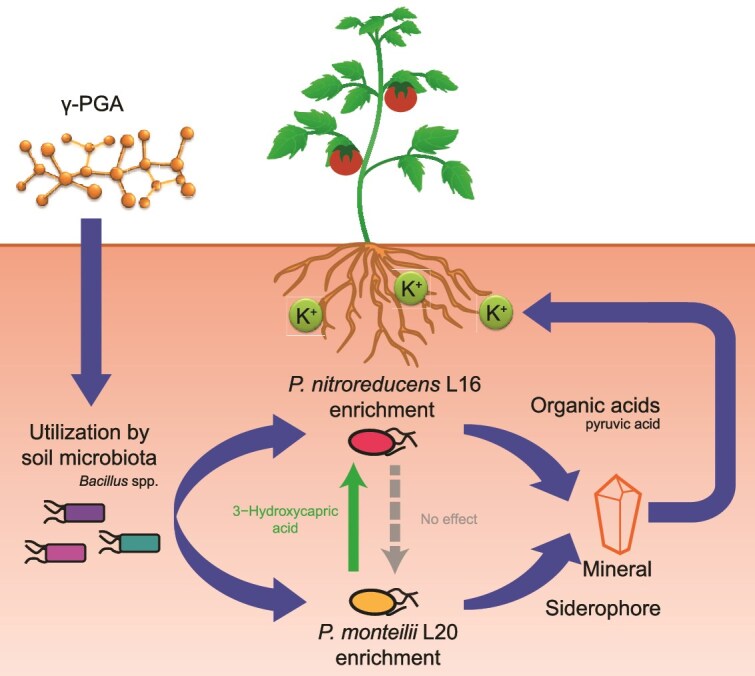

## Introduction

Rhizosphere microbial communities are taxonomically diverse, and as a result, also the metabolic and chemical interactions among them are highly complex [1]. These chemical interactions can be antagonistic, either mediated by resource or interference competition, which have been extensively studied especially in the context of plant pathogen biocontrol [2, 3]. Alternatively, microbial interactions can be facilitative where secretions by one microbe benefit the growth of another microbe via cross-feeding—an interaction which can be commensal or mutualistic [4, 5]. Cross-feeding interactions could have either positive or negative effects on the plant growth by affecting the stability, diversity, and functioning in the rhizosphere microbiomes [6]. A recent study suggests that cross-feeding between the inoculant *Bacillus velezensis* SQR9 and indigenous *Pseudomonas* spp. shaped the microbial community assembly in the cucumber rhizosphere, resulting in enhanced plant protection against salt stress [7]. In contrast, facilitative cross-feeding interactions between the members of tomato rhizosphere resident microbiota were shown to make plants more susceptible to pathogens in another study [8]. Because of instances of conflicting patterns of interaction, we still poorly understand how microbial cross-feeding interactions cascade into plant growth-promotion effects, which limits our ability to manipulate the rhizosphere microbiomes for improved plant productivity.

In addition to mediating the microbial interactions, microbial-derived compounds often stimulate plant growth, mitigate pathogen and abiotic stresses in plants, and break down harmful xenobiotics [9, 10]. As a result, plant growth-promotion can be directly achieved via microbially secreted amino acids, vitamins, phytohormones that benefit plants [11]. Alternatively, microbial secretions can benefit plants indirectly by increasing the bioavailability of limiting key nutrients in the rhizosphere (e.g. organic acids and siderophores) [12, 13]. While the roles of low molecular weight compounds in benefiting plant growth have been extensively studied [14–16], the plant growth effects of more complex, extracellular polymeric substances, and high molecular weight compounds that have different physicochemical (e.g. flocculability and flowability) and biological properties [17, 18] are less well understood. In humans, previous studies have confirmed that microbial extracellular polymeric substances have a variety of beneficial biological activities, including immunomodulatory, anticancer, anti-inflammatory, and hypoglycemic effects, which are thought to be mediated via effects on gut microbial community assembly [19–21]. Complex extracellular polymeric substances could be especially important for triggering cross-feeding interactions, because they are metabolized into smaller molecules that can be used by species that cannot directly metabolize polymeric substances. For example, complex polymers have been reported to underlie a complicated web of microbial interactions dominated by cross-feeding in rumen [22]. However, very little is known about how microbially derived, extracellular polymeric compounds influence the microbe-microbe and microbe-plant interactions in rhizosphere microbiomes.

Here we focused on studying the effects of one such complex substrate, poly-γ-glutamic acid (γ-PGA), on the rhizosphere microbiome composition, functioning and tomato plant growth and nutrient assimilation. Poly-γ-glutamic acid is a nontoxic, water-soluble, and biodegradable extracellular polymeric substance, which is synthesized by a wide range of microbes, including *Bacillus* spp., *Planococcus halophilus*, *Sporosarcina halophile*, *Staphylococus epidermidis*, *Fusobacterium nucleatum*, *Francisella tularensis*, certain archaea and eukaryotes [23–26], through linking D/L-glutamic acid monomers with γ-amide bonds [27, 28]. Microbes produce γ-PGA for surface adhesion and for protection against abiotic and biotic stresses [24, 29–31]. The γ-PGA can also be degraded and used for microbial growth under nutrient starvation [32, 33] and has previously been applied for plant growth-promotion [34–36]. The majority of commercially available γ-PGA is synthesized by *Bacillus* spp. due to safety and high productivity [27, 37]. Although applying γ-PGA-producing bacteria represents a more cost-effective solution for plant growth-promotion than application of pure γ-PGA, the pure γ-PGA has been reported to exhibit higher plant growth-promoting efficiency [36, 38]. Crucially, while γ-PGA has been linked to changes in soil microbiome composition [39] and plant growth [23, 40] via enhanced plant nutrient assimilation and soil nutrient availability [36, 41], the underlying mechanisms are not well understood. Specifically, it remains unclear if the potential plant growth-promoting effects are directly driven by γ-PGA degrading bacteria that use γ-PGA as carbon and nitrogen source [42], or indirectly by other cross-feeding microbes that consume the metabolites and secretions produced by the γ-PGA-degrading bacteria.

Here we studied this experimentally by aiming to answer three main questions: (i) Does γ-PGA promote plant growth via effects on the rhizosphere microbiome composition and functioning? (ii) Are these potential beneficial effects of γ-PGA driven directly by γ-PGA-degrading bacteria or indirectly by other plant-growth-promoting bacteria through cross-feeding? (iii) What are the metabolic interactions and mechanisms underlying plant growth-promotion by γ-PGA? To address these questions, we used multidisciplinary approach where we combined direct plant and laboratory experiments to first identify important plant growth-promoting taxa and then unravel underlying chemical microbe-microbe-plant interactions in a series of validation experiments.

## Materials and methods

### Testing the effects of γ-PGA on plant growth and the rhizosphere microbiome composition

We first conducted a greenhouse experiment to test the effects of γ-PGA on the growth of tomato plants and the rhizosphere microbiome composition. Tomato seeds (*Solanum lycopersicum* cv. Hezuo 903) were surface-sterilized and germinated as described previously [43] before sowing in pots containing 1 kg nursery substrate (soil: sterilized vermiculite = 12:1; w:w; see Appendix S1 for more details of the soil). The same seed variety, seed surface-sterilization and germination procedures, and the soil were used in all experiments throughout this study. The γ-PGA (available from Nanjing Shineking Biological Technology Co., Ltd., Nanjing, China) was dissolved in sterile distilled water and applied via irrigation with final concentrations of 0 (control), 20, 50, 100, 500, and 1000 mg kg^−1^ of nursery substrate before sowing of tomatoes. Each dose treatment of γ-PGA consisted of three independent replicate pots (n = 3) and each pot contained five seedlings. All pots were incubated in a greenhouse with temperature variation ranging between 18 and 26°C, and were replenished daily with sterile distilled water to maintain sufficient water content. The same greenhouse condition was used in all experiments throughout this study. Pots were fertilized with sterile Murashige and Skoog (MS) liquid medium (30 ml) [44] once per week post sowing. Four weeks after sowing, five plants within each pot were destructively harvested and the rhizosphere soil was collected and pooled. To collect rhizosphere soil samples, the excess soil was first removed from the plant roots by shaking and the remaining soil that was closely adhered to the roots (rhizosphere soil) was stored for further analyses. Plant biomass, plant nitrogen (N), phosphorus (P), and potassium (K) contents were determined using auto-Kjeldahl nitrogen analyzer (K9840, Hanon, Shandong, China), ultraviolet spectrophotometer (D-8, Philes, Jiangsu, China), and flame atomic absorption spectroscopy (Varian Spectra AA 220 FS, Victoria, Australia) by following the method described previously [45]. The rhizosphere soil samples were further stored at −80°C for microbiome profiling using sequencing.

To quantify γ-PGA effects on the microbiome composition, rhizosphere soil DNA was extracted using Qiagen PowerSoil DNA extraction kit following the manufacturer's instructions. The V4-V5 region was amplified using the 515F/907R primers [46] with sample-specific barcodes and sequenced on a Miseq System (Illumina; 2 × 250 bp) at Personal Biotechnology Co., Ltd. (Shanghai, China). Raw sequencing data were processed using the UPARSE pipeline (USEARCH v11.0.667) [47] and have been deposited to the NCBI SRA under the accession numbers PRJNA759398 and PRJNA1197718 (see Appendix S2 for more details of microbiome profiling).

### Verifying the role of soil microbiome in γ-PGA-induced plant growth-promotion

We conducted another greenhouse experiment to test how the γ-PGA affects plant growth properties (biomass, N, P, and K contents) in sterilized (control) and unsterilized soils (microbiome present) to distinguish the direct and indirect (via soil microbiome) effects of γ-PGA on plant growth. Surface-sterilized and germinated tomato seeds were sown in culture bottle containing 200 g (dry weight) of autoclaved soil (two cycles of 60 min at 121°C) or natural soil (microbiome present) in the presence or absence of γ-PGA. The γ-PGA was applied with final concentrations of 1000 mg kg^−1^ soil before sowing of tomatoes. Each treatment consisted of four independent replicate culture bottles (n = 4) and each pot contained five seedlings. All culture bottles were fertilized with sterile MS liquid medium (5 ml) once per week post sowing. Five weeks after sowing, five plants within each culture bottle were destructively harvested. Plant biomass, plant N, P, and K contents were determined as described earlier.

### Determining the effects of the γ-PGA-induced microbiome changes on plant growth-promotion

We performed two experiments to test the effects of the γ-PGA-induced microbiome changes on plant growth-promotion. First, we conditioned natural soil with γ-PGA (1000 mg kg^−1^ soil) to drive changes in the microbiome composition in the absence of plants (i.e. γ-PGA-conditioned microbiome; soil conditioning experiment). Soils receiving sterile distilled water were used as controls (i.e. control microbiome; n = 3). The soil was incubated under the same greenhouse condition as described above. Thirty days past the start of the experiment, γ-PGA was undetectable in soil as determined using HPLC analysis with OHpak SB-806 M HQ columns (see below) and all soils were collected for microbiome profiling. The 30-day time point was chosen because our first greenhouse experiment showed that γ-PGA showed clear plant growth-promotion at this stage of the tomato growth cycle. Also, all applied γ-PGA had been degraded by the 30-day time point of soil conditioning experiment, preventing any direct effects of γ-PGA on plant growth or microbiome functioning during the transplantation experiments; all effects were hence driven by transplanted microbes. However, one limitation of choosing the 30-day time point is that we might have lost some transient microbiome changes that potentially vanished once the γ-PGA was depleted.

In the second experiment, we compared the effects of the γ-PGA-conditioned microbiome changes and control microbiomes on plant growth-promotion by conducting a microbiota transplant experiment. Briefly, soils were collected from soil conditioning experiment and replicates (n = 3) were kept independent for each treatment. To prepare microbial inoculum, the collected soil from each independent sampling replicate was mixed with 50% sterile MS medium at a ratio of 1:2 (w:v) on an orbital shaker at 120 rpm for 30 min at room temperature. After 1 hour of setting, microbes were separated into a supernatant fraction by centrifuging at 800 × g for 5 min. The resulting supernatants were used as the microbial inocula. Microbial inocula of 70 ml were then added to tissue culture bottles containing 200 g (dry weight) of autoclave-sterilized soil (two 60 min cycles at 121°C). No fertilizer was applied in addition to microbial inoculum. Five surface-sterilized and germinated seeds were sown into each tissue culture bottle following the transplantation of microbial inoculum. Five weeks after sowing, the plants and the rhizosphere soils of five plants within a replicate culture bottle were collected and pooled. Plant biomass, plant N, P, and K contents were determined and the rhizosphere bacterial community composition was profiled as described earlier. Total bacterial abundance in the rhizosphere was determined based on 16S rRNA gene copy number using quantitative PCR analysis with primer set Eub338-Eub518 [48]. Each individual sample was measured in triplicate on a 7500 Fast System (Applied Biosystems, CA, USA) using the SYBR Premix Ex Taq Kit (Takara, Dalian, China).

### Identification of rhizobacteria that were enriched by γ-PGA treatments

To identify the rhizobacteria enriched by γ-PGA, we compared the rhizosphere bacterial community composition of the γ-PGA-conditioned microbiome and control microbiome treatments after the microbiota transplant experiment. We found 11 zero-radius operational taxonomic units (zOTUs) that were enriched after treatment with the γ-PGA-conditioned microbiome (top 15% zOTUs with highest average relative abundances in both control and γ-PGA-conditioned microbiota transplant treatments with *P* value <0.001). The average relative abundances of the top 15% zOTUs ranged between 0.2% and 11.6%, adding up to 65.3% of total sequence abundance. To obtain culturable bacteria of these 11 zOTUs, we isolated bacteria from the rhizosphere soil samples of the γ-PGA-conditioned microbiome treatment after the microbiota transplant experiment. To maximize the isolation success, diluted soil suspensions were plated on four agar plates including Luria Bertani (LB), nutrient agar (NA), tryptic soy agar (TSA), and Gause NO. 1 agar. All the isolates with different morphologies were selected, resulting in 33–68 isolates for each of the four agar plates and a total of 173 isolates. These bacterial isolates were then taxonomically classified based on the full-length 16S rRNA gene sequences, which were amplified using the primer set F27 and R1492 [49] and further sequenced using Sanger sequencing with a previously published methodology [50]. Twelve of the 173 isolates were removed from further analysis because of potential clonal duplicates (100% sequence similarity), resulting in 161 unique rhizobacteria. To match the full-length 16 s rRNA gene sequences of the bacterial isolates with the zOTU sequences obtained from high throughput sequencing, we trimmed the full-length 16S rRNA gene sequences of the isolates to the same region of the high-throughput sequencing data using UPARSE [47]. Sequences were then mapped to the zOTU sequences at the threshold of 100% similarity, score of 370, and e value of 1e^−150^ using UPARSE. We found four isolates/zOTUs enriched in the γ-PGA-conditioned microbiome treatment (top 15% zOTUs with *P* < .001) after matching 16S rRNA gene sequences of the isolates with the zOTU data.

As the microbiota transplant experiment demonstrated that γ-PGA increased plant K content compared to no-γ-PGA control treatment, we focused on K-solubilizing bacteria among all the 161 unique rhizobacteria, including the four isolates with matched zOTUs enriched in the γ-PGA-conditioned microbiome treatment. We used Aleksandrov medium with K-feldspar powder as the sole source of K [51] to qualitatively and quantitatively test the K-solubilization ability of these isolates (see Appendix S3 for more details).

Of the four isolates with matched zOTUs in the γ-PGA-conditioned microbiome treatment, only strain L20 (zOTU_7, *Pseudomonas monteilii*, GenBank: OP278965) and strain L16 (zOTU_17, *Pseudomonas nitroreducens*, GenBank: OP278966) showed K-solubilizing ability in both qualitative and quantitative tests. We next inoculated overnight-grown bacterial suspension to tomato seedlings to determine the effects of *P. nitroreducens* L16 and *P. monteilii* L20 on plant growth-promotion (see Appendix S4 for more details). The strains *P. nitroreducens* L16 and *P. monteilii* L20 were cultivated in LB liquid medium on an orbital shaker (120 rpm) at 30°C. Overnight-grown bacteria (early exponential phase) were harvested by centrifugation (10 000 × g for 6 min), washed with sterile saline solution (0.9% NaCl), and adjusted to appropriate concentrations using dilution or concentration based on their optical density (OD_600_) as previously reported [14, 52]. As a result, all the used microbial inocula were at similar stages of bacterial growth (early exponential phase) despite different densities. The same bacterial inocula preparation methods were used in all experiments throughout this study.

### Determining the role of organic acids in K-solubilization

Potassium-solubilizing bacteria often dissolve K minerals by secreting organic acids which are associated with acidolysis and complexolysis exchange reactions [53]. To determine the organic acids secreted by *P. nitroreducens* L16 and *P. monteilii* L20, HPLC analyses were performed using a Shimadzu LC-20A system (Shimadzu, Kyoto, Japan) based on a previously published methodology [54].

To verify the role of organic acids in K-solubilization of *P. nitroreducens* L16 and *P. monteilii* L20, 5% bacterial suspension (OD_600_ = 0.5) was inoculated to liquid Aleksandrov medium, which was unbuffered (pH 4.0 for *P. nitroreducens* L16 culture medium and pH 4.3 for *P. monteilii* L20 culture medium) or buffered to pH 7 with 100 mM 2-(N-morpholino) ethanesulfonic acid (MES) [55]. Aleksandrov medium receiving 5% sterile distilled water was used as a control (n = 3). After 9 days of incubation, the soluble K content in supernatants was determined using Optima 8000 ICP-OES (Perkin Elmer, USA).

### Determining the role of siderophores in K-solubilization

The mineral weathering potential of rhizobacteria can also be linked with production of chelates like siderophores [56], and as a result, we determined the siderophore production by *P. nitroreducens* L16 and *P. monteilii* L20 in iron-limited MKB medium (siderophore production is induced in iron-limited condition) [57] using CAS assay [58] (see Appendix S5 for more details).

To verify whether *P. nitroreducens* L16 and *P. monteilii* L20 produce siderophores to chelate and solubilize K, we collected supernatants from bacteria grown under four different treatments after 48 h of growth in modified MKB medium (K_2_HPO_4_ was changed to Na_2_HPO_4_): (i) sterile iron-limited MKB medium (negative control); (ii) iron-limited MKB medium inoculated with *P. nitroreducens* L16 or *P. monteilii* L20 (test for K-chelation by siderophores); (iii) iron-rich MKB medium (MKB medium supplemented with 50 μM FeCl_3_) inoculated with *P. nitroreducens* L16 or *P. monteilii* L20 (under iron-rich condition, bacteria produce few siderophores but other compounds are secreted); (iv) iron-limited MKB medium inoculated with *P. nitroreducens* L16 or *P. monteilii* L20, which were further replenished with 50 μM FeCl_3_ (the supernatant treatment still contains siderophores but are invalidated for iron uptake, as the iron is available in excess [59]. We then added 2 g L^−1^ sterile K-feldspar powder to these four supernatants. After 9 days of incubation, the soluble K content in the supernatants was determined as described earlier.

### Utilization of γ-PGA by *P. nitroreducens* L16 and *P. monteilii* L20

To examine whether the *P. nitroreducens* L16 and *P. monteilii* L20 can utilize γ-PGA, 1% bacterial suspension (OD_600_ = 2.5) was added to OS minimal medium [60] supplemented with 1 g L^−1^ γ-PGA as the sole carbon source. OS minimal medium supplemented with only 1% bacterial suspension (OD_600_ = 2.5) was included as a control (n = 3) and cell growth was determined at OD_600_.

We also conducted HPLC analysis to determine the remaining γ-PGA in OS medium 24 h after inoculation of *P. nitroreducens* L16 or *P. monteilii* L20. Briefly, separation was performed using a Shimadzu LC-20A system (Shimadzu, Kyoto, Japan) with two OHpak SB-806 M HQ columns (8.0 mm × 300 mm, Shodex, Japan). The solvent system consisted of Na_2_SO_4_ and acetic acid (pH 4.0). The UV–visible photodiode detector was set to 210-nm wavelength. 0.2, 0.4, 0.6, 0.8, 1.0, and 1.2 g L^−1^ γ-PGA were included as standards.

### Effects of the metabolites secreted by γ-PGA-utilizing rhizobacteria on the growth of *P. nitroreducens* L16 and *P. monteilii* L20

As neither *P. nitroreducens* L16 nor *P. monteilii* L20 could utilize γ-PGA, we tested if other isolated rhizobacteria (n = 159; 161 unique isolates other than *P. nitroreducens* L16 and *P. monteilii* L20) could utilize γ-PGA and if their metabolites could affect the growth of *P. nitroreducens* L16 and *P. monteilii* L20. This test was done by growing the 159 isolates in OS minimal medium supplemented with 1 g L^−1^ γ-PGA as the sole carbon source as described earlier and then extracting isolate supernatants to observe their effects on the growth of *P. nitroreducens* L16 and *P. monteilii* L20 (see Appendix S6 for more details).

### Testing the coexistence and cross-feeding between *P. nitroreducens* L16 and *P. monteilii* L20 isolates

To determine whether *P. nitroreducens* L16 and *P. monteilii* L20 constituted stable consortia, we inoculated 1% mixture of bacterial suspension of *P. nitroreducens* L16 and *P. monteilii* L20 (OD_600_ = 5 for both strains) to liquid LB medium (*P. nitroreducens* L16 and *P. monteilii* L20 grow well in LB medium) and measured species coexistence with different inoculation ratios of *P. nitroreducens* L16 and *P. monteilii* L20: 999:1, 499:1, 199:1, 99:1, 9:1, 1:1, 1:9, 1:1, 1:9, 1:99, 1:99, 1:199, 1:499, and 1:999 (v:v); each inoculation ratio was replicated three times (n = 3). The frequencies of *P. nitroreducens* L16 and *P. monteilii* L20 were determined by serial dilution plating based on colony morphology differences on LB plates (The colonies of *P. monteilii* L20 are circular and light yellow with erose margin, whereas the colonies of *P. nitroreducens* L16 are irregular and white with undulate margin).

To test metabolic interactions (potential antagonism and facilitation) between *P. nitroreducens* L16 and *P. monteilii* L20, we grew them in the presence of each other’s supernatants (see Appendix S7 for more details).

### Identification of growth-promoting metabolites of *P. nitroreducens* L16 secreted by *P. monteilii* L20

To identify the growth-promoting metabolites of *P. nitroreducens* L16 secreted by *P. monteilii* L20, we first inoculated 2% bacterial suspension of *P. monteilii* L20 (OD_600_ = 0.5) to OS minimal medium supplemented with 4 g L^−1^ glucose as the sole carbon source. The concentration of glucose was determined every 12 h using SBA-40C biosensor (Jinan, China). After incubation for 3 days, *P. monteilii* L20 utilized all the glucose. The culture medium was then collected and filtered to obtain the metabolites of *P. monteilii* L20. We then inoculated 2% bacterial suspension of *P. nitroreducens* L16 (OD_600_ = 0.5) to metabolites of *P. monteilii* L20. Metabolites of *P. monteilii* L20 receiving 2% sterile distilled water were used as controls (n = 4). After incubation for 3 days, the medium was filtered to remove bacterial cells, lyophilized, and re-dissolved in 30% methanol (v: v) (lyophilized powder:solvent = 1:50; w:v). A 20 μL aliquot of each sample was injected for UHPLC–MS/MS analysis (see Appendix S8 for more details) and the metabolite raw data are provided as External Database S1. The metabolites which were abundant in the supernatant of *P. monteilii* L20, but significantly decreased when cultivated in the presence of *P. nitroreducens* L16, were identified as potential *P. nitroreducens* L16-promoting compounds secreted by *P. monteilii* L20. The effects of three potential *P. nitroreducens* L16 growth-promoting compounds (glyceraldehyde, L-2-hydroxyglutaric acid, and 3-hydroxycapric acid) were further tested by using their chemical standards as sole carbon source in OS minimal medium.

### Statistical analyses

A factorial analysis of variance (ANOVA, Tukey's HSD test) and Student’s *t* test were carried out to compare mean differences between the treatments. The Shannon index was calculated using Mothur (v.1.46.1) [61]. Differences in the composition of bacterial communities were assessed based on unweighted UniFrac or Bray-Curtis distances using Mothur. Statistical significance of the differences in bacterial community composition between the treatments was compared using analysis of molecular variance (AMOVA). The plant growth traits (biomass, N, P, and K contents) were first standardized using the Z-score transformation (scale command in R), after pairwise plant growth trait dissimilarities were calculated based on Z-scores using Euclidean distance (vegdist command in the vegan R package). Discriminating taxa between the treatments were identified using the linear discriminant analysis effect size (LEfSe) method [62] at the threshold of linear discriminant analysis (LDA) score of 2 and *P* < .05. The phylogenetic tree file was generated using FastTree and visualized using iTOL [63]. All analyses were conducted using R and SPSS (v. 22).

## Results

### Application of γ-PGA increases the K content in tomato plants and alters the rhizosphere microbiota composition

**Figure 1:**
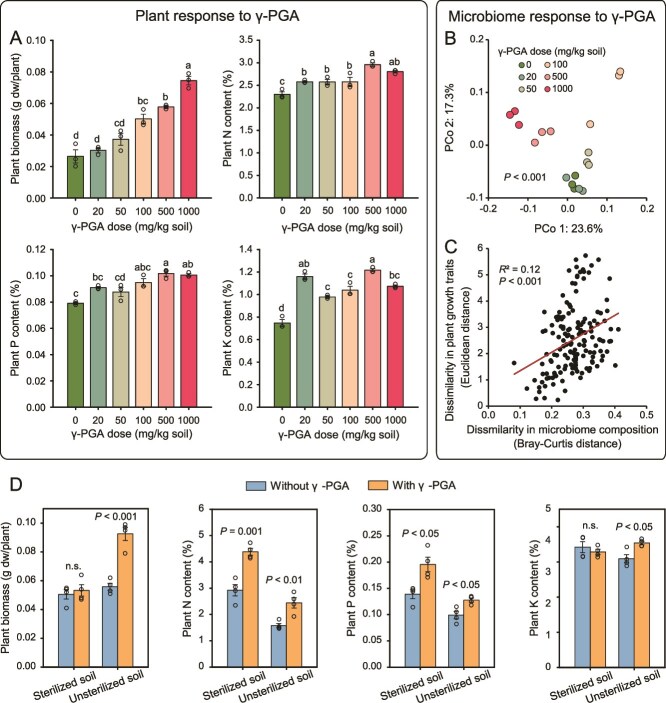
**γ-PGA promotes plant growth by shaping the rhizosphere microbiome composition.** (**A**) the effects of different doses of γ-PGA on plant biomass, nitrogen (N), phosphorus (P), and potassium (K) contents in tomato. (**B**) Rhizosphere bacterial community compositions clustered based on the application dose of γ-PGA. (**C**) Positive relationship between the dissimilarity of rhizosphere bacterial communities and plant growth traits. (**D**) Effects of γ-PGA (1000 mg kg^−1^ soil) on plant biomass, N, P, and K contents of tomato in sterilized soil and unsterilized soil (microbiome present).In panel B, the percentages of explained variation are based on unweighted UniFrac distances on both axes. In panels A and D, error bars indicate means ± SE.

We first conducted a greenhouse experiment to test the effects of γ-PGA application on plant growth, nutrient assimilation and the rhizosphere microbiome composition. We found that γ-PGA application increased the biomass (*F*_5,12_ = 39.3, *P* < .001, ANOVA), and phosphorus (P; *F*_5,12_ = 16.1, *P* < .001), nitrogen (N; *F*_5,12_ = 16.5, *P* < .001), and potassium (K; *F*_5,12_ = 55.3, *P* < .001) contents in tomato plants (Fig. 1A), with 500 mg kg^−1^ and 1000 mg kg^−1^ of γ-PGA treatments showing the clearest effects. Specifically, 1000 mg kg^−1^ of γ-PGA increased the biomass, N, P, and K contents in tomato plants by 183.5%, 21.8%, 27.8%, and 43.6%, respectively. Although the γ-PGA application did not have significant effects on the rhizosphere bacterial diversity (Shannon index; F_5,12_ = 2.0, *P* = .16, ANOVA; Fig. S1A), it clearly changed the microbiome composition (*P* < .001, AMOVA; Fig. 1B and S1B) and higher γ-PGA concentrations were associated with greater changes (Fig. S1C). There was a significant correlation (*R* = 0.35, *P* < .001; Fig. 1C) between the pairwise dissimilarity in plant growth traits (biomass, N, P, and K contents; Euclidean distance) and pairwise dissimilarity in the rhizosphere bacterial community composition (Bray-Curtis distance), suggesting γ-PGA may potentially promote plant growth by affecting the rhizosphere microbiome composition and functioning.

To distinguish the direct and indirect (via soil microbiome) effect of γ-PGA on plant growth, we tested plant growth properties in sterilized and unsterilized soil in the presence and absence of γ-PGA (1000 mg/kg). Similar to previous results, we found that γ-PGA significantly increased the biomass and N, P, and K contents of tomato plants in unsterilized soil (*P* < .05, Student’s *t* test; Fig. 1D and S2). Furthermore, we also found that γ-PGA significantly increased N (*P* = .001, Student’s *t* test) and P (*P* = .013) contents of tomato plants in sterilized soil by 50.0% and 40.9%, respectively, but did not have significant effects on tomato biomass (*P* = .61) or K content (*P* = .47). These results suggest that γ-PGA could directly increase plant N and P content in the absence of microbiota but that the increase in plant biomass and K content was indirectly mediated via effects on soil microbiota.

**Figure 2:**
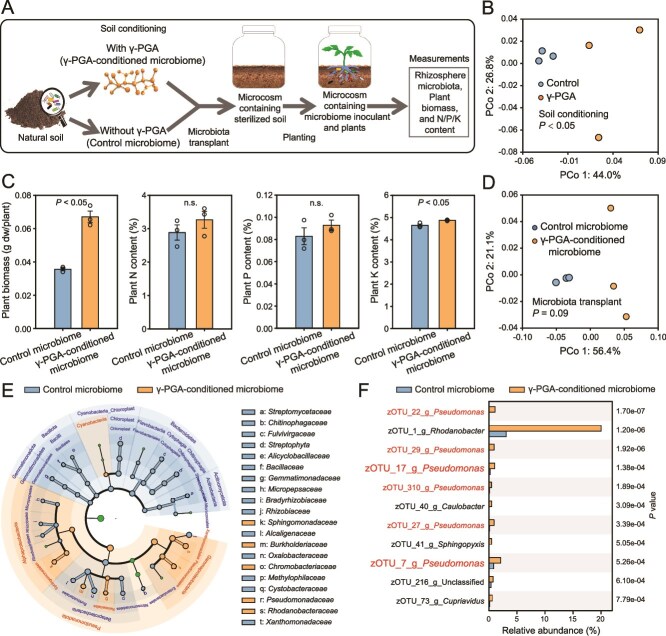
**The effects of γ-PGA-conditioned microbiome on the plant biomass, P, N, K contents in tomato, and rhizosphere bacterial community composition.** (**A**) Schematic representation of the soil conditioning and microbiota transplant experiment. (**B**) Effects water (control) of γ-PGA (1000 mg kg^−1^ soil) conditioning on soil bacterial community composition. (**C**) Effects of γ-PGA-conditioned microbiome on plant biomass, P, N, and K contents in tomato at the end of microbiota transplant experiment. (**D)** Effects of γ-PGA-conditioned microbiome on the rhizosphere bacterial community compositions at the end of microbiota transplant experiment. (**E**) LEfSe cladogram showing the bacterial taxa (highlighted by small circles and shading, relative abundance >0.1%) of statistically differences (LDA score > 2, *P* < .05) between control and γ-PGA-conditioned microbiota transplant treatments. (**F**) Top zOTUs (15%) significantly enriched (*P* < .001) in the rhizosphere by γ-PGA-conditioned microbiota transplant relative to control treatment. In panels B and D, the percentages of explained variation are based on unweighted UniFrac distances on both axes. In panel C, error bars indicate means ± SE. In panel E, blue and orange colors represent taxa that were abundant in control and γ-PGA-conditioned microbiome, whereas green color represents non-significant taxa. Each circle’s diameter is proportional to the taxon’s abundance. In panels C and F, *P*-values were calculated using Student’s *t* test. In panel F, zero-radius OTUs assigned to *Pseudomonas* spp. are highlighted in red. Zero-radius OTU_7 and zOTU_17 are highlighted on larger font.

To further verify the causality between the γ-PGA-induced microbiome changes and plant growth-promotion, we first conditioned natural soil with γ-PGA and quantified changes in microbiome composition in the absence of plants (“γ-PGA-conditioned microbiome”) (soil conditioning experiment; Fig. 2A). Soils without γ-PGA application (“control microbiome” receiving sterile water only) were set up as controls. When the γ-PGA in soil was undetectable and had been used up or broken down, we compared the effects of γ-PGA-conditioned and control microbiomes on plant growth in a separate laboratory microbiota transplant experiment (See methods and Fig. 2A). We found that γ-PGA had significant on soil bacterial community composition (*P* = .015; AMOVA) at the end of the soil conditioning experiment (Fig. 2B). Specially, 73 zOTUs were enriched by γ-PGA application and most of these zOTUs (39/73) belonged to *Variovorax*, *Pseudomonas*, *Sphingomonas*, *Caulobacter*, and *Flavisolibacter* genera (top 15% with *P* < .05; Fig. S3). Although the γ-PGA-conditioned microbiome did not have significant effect on N (*P* = .32, Student’s *t* test) or P contents (*P* = .32) in tomato plants at the end of microbiota transplant experiment, it significantly increased the tomato biomass (*P* = .001) and K contents (*P* = .021) by 87.9% and 4.9%, respectively, compared to the control microbiome treatments (Fig. 2C). Moreover, whereas the γ-PGA-conditioned microbiota transplant did not have significant effect on the total bacterial abundances (Fig. S4A), and had only tendency to change the beta-diversity of rhizosphere bacterial community relative to the control treatments (control microbiome versus γ-PGA-conditioned microbiome, *P* = .09, AMOVA; Fig. 2D), we found clear clustering of transplanted bacterial community compositions between the γ-PGA-conditioned and the control microbiome treatments based on unweighted UniFrac (Fig. 2D) and Bray-Curtis distances (Fig. S4B). Specifically, the application of γ-PGA-conditioned microbiome transplant significantly increased the relative abundances of *Pseudomonadota* phylum, and *Sphingomonadaceae*, *Burkholderiaceae*, *Chromobacteriaceae*, *Pseudomonadaceae*, *Rhodanobacteraceae* families, whereas it decreased the relative abundances of *Gemmatimonadota*, *Bacillota*, *Cyanobacteria*_*Chloroplast, Bacteroidetes*, and *Actinomycetota* phyla, and 15 bacterial families (LDA score > 2, *P* < .05, LEfSe analysis; Fig. 2E). Eleven top bacterial zOTUs enriched by the γ-PGA-conditioned microbiota transplant included six *Pseudomonas* species, one *Rhodanobacter*, one *Caulobacter*, one *Cupriavidus*, and one unclassified zOTU species (top 15% with *P* value <0.001; Fig. 2F), whereas 10 top bacterial zOTUs that were reduced due to the γ-PGA-conditioned microbiota transplant included two *Pseudomonas* species, two *Sphingomonas* species, one *Pseudoduganella*, one *Massilia*, one *Azohydromonas*, one *Lysobacter*, one *Luteimonas*, and one *Streptophyta* (top 15% with *P* value <0.001; Fig. S4C). The 11 zOTUs enriched by γ-PGA-conditioned microbiota transplant (Fig. 2F) were often detected in the first greenhouse experiment (γ-PGA dose experiment; Fig. S5A) and the soil conditioning experiment (Fig. S5B). For example, the relative abundance of zOTU_1, zOTU_17, and zOTU_40 were significantly correlated with γ-PGA dose in the first greenhouse experiment and were significantly enriched by soil conditioning with γ-PGA. Similarly, zOTU_7, zOTU_22, zOTU_27, and zOTU_29 were enriched by one of the γ-PGA dose treatments in the first greenhouse experiment and were significantly enriched by soil conditioning with γ-PGA. Moreover, all the *Pseudomonas* zOTUs that were enriched by soil conditioning with γ-PGA showed higher relative abundances in the rhizosphere of γ-PGA-conditioned rhizosphere microbiomes compared to water-conditioned, control transplants (Fig. S3). Together, these results suggest that γ-PGA application can increase plant K content and plant biomass likely by altering rhizosphere microbiome community composition and potential functioning.

### γ-PGA promotes plant growth by enriching two potassium-solubilizing *pseudomonas* strains

**Figure 3:**
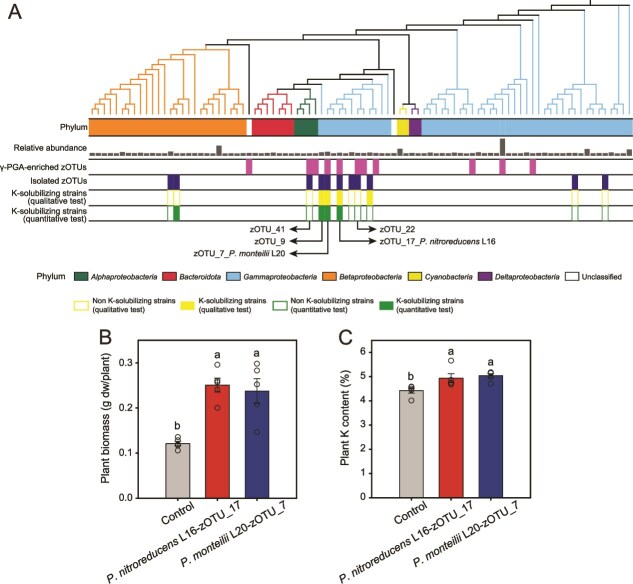
**Identification of the potassium-solubilizing bacteria enriched by γ-PGA-conditioned microbiota transplant.** (**A**) Phylogenetic tree of top zOTUs in the rhizosphere at the end of the microbiota transplant experiment. The first strip indicates the phylum affiliation of the top 15% zOTUs in control and γ-PGA-conditioned microbiota transplant treatments. The grey bars (the second strip) indicate the average relative abundance of each zOTUs (0.2%–11.6% in relative abundance with 65.3% of total sequence abundance) in the rhizosphere of control and γ-PGA-conditioned microbiota treatments. The third strip indicates the isolated zOTUs which were significantly enriched by γ-PGA-conditioned microbiota transplant based on amplicon sequencing data. The fourth strip indicates the isolated zOTUs. The fifth and the sixth strips indicate the K-solubilizing ability of isolated zOTUs based on qualitative and quantitative tests, respectively. (**B**) and (**C**) Effects of *Pseudomonas nitroreducens* L16 (zOTU_17) and *Pseudomonas monteilii* L20 (zOTU_7) on biomass and K content of tomato plants, respectively. In panels B and C, error bars indicate means ± SE.

To validate the plant growth-promoting effects by bacteria enriched in the γ-PGA-conditioned microbiome, we isolated 161 unique bacterial strains from these treatment samples at the end of the microbiota transplant experiment. We were able to obtain four isolates (zOTU_7, zOTU_17, zOTU_22, and zOTU_41; mapping threshold: 100% similarity, score of 370, and e value of 1e^−150^) that belonged to the same 11 enriched zOTUs detected in the γ-PGA-conditioned microbiome treatment (Fig. 2F and3A). As our greenhouse experiment (Fig. 1D) and microbiota transplant experiment (Fig. 2C) demonstrated increase in plant K assimilation, we screened these isolates for their K-solubilizing ability. Of the four isolates, the strains L20 (zOTU_7, GenBank: OP278965) and L16 (zOTU_17, GenBank: OP278966) showed high K-solubilizing ability in both qualitative and quantitative tests (Fig. 3A and S6). The isolate L20 was the most similar to *P. monteilii* CIP 104883 strain (percent identity 99.9%; e value 0.0), whereas the isolate L16 was most similar to *P. nitroreducens* NBRC 12694 strain (percent identity 99.2%; e value 0.0) (Fig. S7). Another greenhouse experiment was conducted to demonstrate that both *P. nitroreducens* L16 and *P. monteilii* L20 bioinoculants could increase the biomass and K content of tomato (Fig. 3B and  3C). Specifically, *P. nitroreducens* L16 and *P. monteilii* L20 increased the tomato biomass by 106.9% and 95.9%, respectively, and increased the plant K content by 11.5% and 2.1%, respectively. While it is possible that other bacteria could have had a positive effect on plant growth-promotion (e.g. zOTU_9 most similar to *Enterobacter ludwigii* EN-119, which showed strong K-solubilizing ability), they were not significantly enriched in the γ-PGA-conditioned microbiota transplant treatment (Fig. 3A). As a result, the two *Pseudomonas* taxa were likely relatively more important in explaining the plant growth-promoting effect by γ-PGA supplementation, and hence, we next focused on the exact mechanisms of K-solubilization by these two *Pseudomonas* strains (L16 and L20).

### Mechanism of K-solubilization by *P. nitroreducens* L16 and *P. monteilii* L20

**Figure 4:**
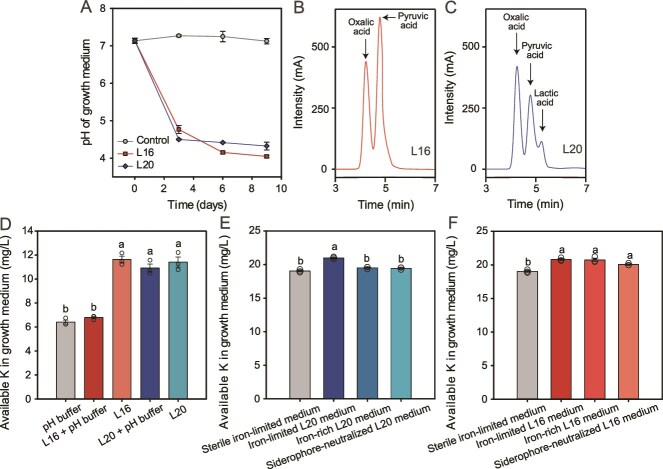
**Potassium-solubilizing mechanisms of the bacteria enriched by γ-PGA.** (**A**) Culture medium pH during the growth of the K-solubilizing bacteria enriched by γ-PGA (*Pseudomonas nitroreducens* L16 and *Pseudomonas monteilii* L20). (**B**) and (**C**) Chromatograms of organic acids secreted by *P. nitroreducens* L16 and *P. monteilii* L20, respectively. (**D**) Available K content in culture medium unbuffered or buffered to pH 7. (**E**) and (**F**) Available K content in culture medium of *P. nitroreducens* L16 and *P. monteilii* L20, respectively, when siderophores were not produced (iron-rich medium) or were neutralized (siderophores-neutralized medium). In panels A, D, E, and F, error bars indicate means ± SE.

As K-solubilization by bacteria is often attributed to acidification, we first tested if *P. nitroreducens* L16 and *P. monteilii* L20 changed the pH of their growth media and if this was associated with K-solubilization. We found that the pH of the spent growth media clearly decreased especially by *P. nitroreducens* L16 and in lesser degree by *P. monteilii* L20, indicative of acidification (Fig. 4A). The decrease in pH was due to the production of oxalic and pyruvic acids by *P. nitroreducens* L16 and the production of oxalic, pyruvic, and lactic acids by *P. monteilii* L20 (Fig. 4B and  4C). The growth of *P. nitroreducens* L16 led to increase in the levels of available K in the growth medium, and this effect was significantly decreased when the growth medium was buffered to pH 7, preventing the acidification (L16 versus L16 + pH buffer, *P* < .001; Tukey's post hoc test; Fig. 4D). This suggests that the production of organic acids by *P. nitroreducens* L16 aided in K-solubilization by acidifying the surrounding environment and releasing bound K ions from mineral K. In contrast, buffering the growth media did not have significant effects on K-solubilization efficiency by *P. nitroreducens* L20 (*P* = .74; Tukey's post hoc test; Fig. 4D), which indicates that organic acid production was not the key mechanism behind K-solubilization by this bacterial species. Instead, we found that *P. monteilii* L20 could produce iron-chelating siderophores (Fig. S8A), and that the production of siderophores significantly correlated with the increase in K availability in the growth medium (*R* = 0.69, *P* = .013; Fig. S8B). When the siderophores were neutralized using excess (50 μM) FeCl_3_ (iron-limited L20 medium versus siderophore-neutralized L20 medium, *P* < .001, Tukey's post hoc test; Fig. 4E), or when siderophores were not produced due to excess of iron present in the growth medium (iron-limited L20 medium versus iron-rich L20 medium, *P* < .001), no K solubilization was observed. In contrast, *P. nitroreducens* L16 retained its ability to solubilize K under both of these siderophore manipulation conditions (iron-limited L16 medium versus siderophores-neutralized L16 medium, *P* = .07; iron-limited L16 medium versus iron-rich L16 medium, *P* = 1.0; Tukey's post hoc test; Fig. 4F). Together, these results demonstrate that *Pseudomonas* species used different strategies for K-solubilization: *P. nitroreducens* L16 solubilized K mainly through acidification, whereas *P. monteilii* L20 solubilized K by secreting siderophores as chelating agents.

### γ-PGA indirectly enriches both K-solubilizing *pseudomonas* strains by stimulating microbiota especially *bacillus* species

**Figure 5:**
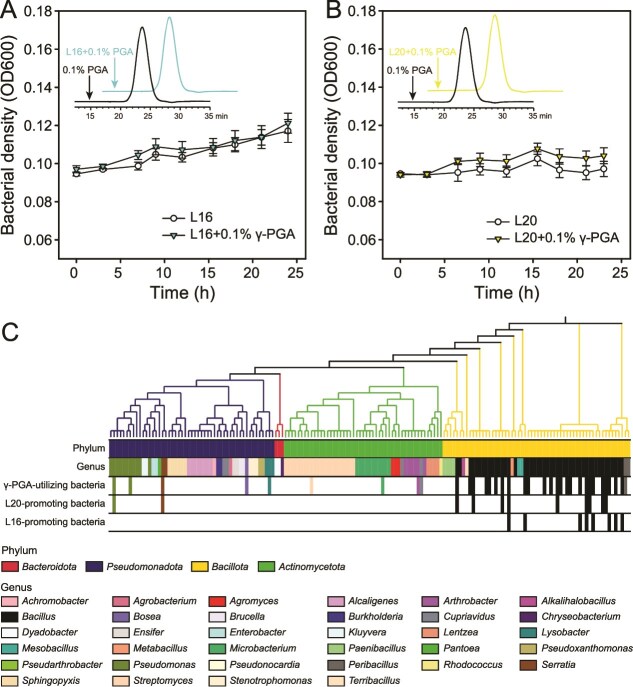
**γ-PGA increased *Pseudomonas***  ***nitroreducens* L16 and *Pseudomonas monteilii* L20 densities by stimulating *Bacillus* spp. consortium.** (**A**) and (**B**) effects of γ-PGA on growth of *P. nitroreducens* L16 and *P. monteilii* L20, respectively. In panels a and B, chromatograms indicate the peaks of γ-PGA determined by HPLC. **(C)** Phylogenetic tree of all the isolates in tomato rhizosphere of microbiota transplant experiment. The first and second strips indicate the phylum and the genus affiliation of the isolates, respectively. The third, fourth, and fifth strip indicate γ-PGA-utilizing isolates, L20-promoting isolates, and L16-promoting isolates, respectively (labeled according to Fig. S10 and S11). We define growth promotion as a significant increase (*P* < .05, Student’s *t* test) in OD_600_ of the isolated rhizobacteria in the presence of γ-PGA or bacterial metabolites relative to negative control treatments (no γ-PGA or bacterial metabolites). In panels A and B, error bars indicate means ± SE.

To test if γ-PGA promoted the growth of *P. nitroreducens* L16 and *P. monteilii* L20 directly, we grew both species in conditions with γ-PGA as the sole carbon source and quantified the γ-PGA content in the spent medium. We found that neither *P. nitroreducens* L16 nor *P. monteilii* L20 strains could utilize γ-PGA for their own growth (Fig. 5A, 5B, and S9), which suggests that they must have benefitted from γ-PGA application indirectly. To test if these indirect benefits were mediated, we measured the growth of other isolated microbial taxa with γ-PGA as the sole carbon source. We found that γ-PGA significantly promoted the growth of 35 of the unique 161 rhizobacteria (*P* < .05; Student’s *t* test) by 5.9%–177.5%. Crucially, 71.4% of the γ-PGA-utilizing isolates (25/35 isolates) belonged to *Bacillus* genus (Fig. 5C and S10). Of these γ-PGA-utilizing isolates, 11 (9/11 *Bacillus*) promoted the growth of *P. monteilii* L20 and eight (all *Bacillus*) promoted the growth of *P. nitroreducens* L16 (Fig. 5C andS11), and the growth of both *Pseudomonas* species were promoted in similar degree by the *Bacillus* species (Fig. S11). Together, these results suggest that γ-PGA indirectly promoted the growth of K-solubilizing *Pseudomonas* species by promoting the growth of *Bacillus* species, which in turn produced metabolites that could be utilized by *Pseudomonas* species.

### *P. nitroreducens* L16 and *P. monteilii* L20 strains form compositionally stable cocultures with high K-solubilization efficiency due to intra-species cross-feeding

**Figure 6:**
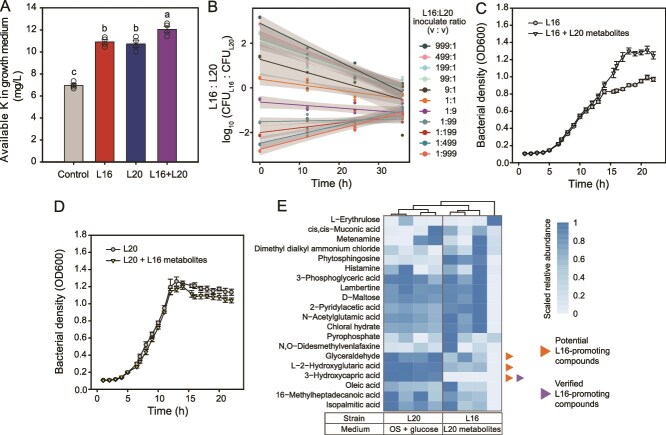
**Metabolic interactions between *Pseudomonas***  ***nitroreducens* L16 and *Pseudomonas monteilii* L20.** (**A**) Joint K solubilization efficiency of *P. nitroreducens* L16 and *P. monteilii* L20. (**B**) Convergence of the ratio of the abundance of *P. nitroreducens* L16 and *P. monteilii* L20 with different initial inoculation ratios. (**C**) Effects of the metabolites of *P. monteilii* L20 on the growth of *P. nitroreducens* L16. (**D**) Effects of the metabolites of *P. nitroreducens* L16 on the growth of *P. monteilii* L20. (**E**) Identification of metabolites modulating the interactions between *P. nitroreducens* L16 and *P. monteilii* L20. Glyceraldehyde, L-2-hydroxyglutaric acid, and 3-hydroxycapric acid were identified as potential *P. nitroreducens* L16-promoting compounds secreted by *P. monteilii* L20, as they were abundant in the spent medium (OS+glucose) of *P. monteilii* L20, and were significantly reduced from the supernatants by *P. nitroreducens* L16. The cross-feeding effects of one of candidates (3-hydroxycapric acid) was experimentally verified using chemical standards (i.e. verified L16-promoting compounds). In panels A, C, and D, error bars indicate means ± SE.

As *P. nitroreducens* L16 and *P. monteilii* L20 solubilized K through different processes, we tested their joint K-solubilization efficiency in co-cultures. We found that the joint K-solubilization efficiency of *P. nitroreducens* L16 and *P. monteilii* L20 was 8.2% and 12.4% higher than *P. nitroreducens* L16 (*P* = .022; Tukey's post hoc test) or *P. monteilii* L20 (*P* = .008) grown alone (Fig. 6A). These *Pseudomonas* species also formed stable consortia and their final co-culture ratios converged to 1:1 regardless of the initial inoculum ratios that ranged from 999:1 to 1:999 (Fig. 6B and S12). To understand the consortia stability in more detail, we explored if these two species also engaged in cross-feeding interactions that were potentially mutually beneficial. We found that the growth of *P. nitroreducens* L16 was clearly enhanced by the metabolites of *P. monteilii* L20 (Fig. 6C) and *P. monteilii* L20 was found to induce swarming behavior to attract *P. nitroreducens* L16 (Fig. S13). The OD_600_ value of *P. monteilii* L20 was slightly lower in the presence of metabolites produced by *P. nitroreducens* L16, but this decrease was not statistically significant (Fig. 6D). As a result, only commensal interaction was observed between *P. nitroreducens* L16 and *P. monteilii* L20. To better understand the key metabolites involved, metabolomics analysis was conducted. We found that glyceraldehyde, L-2-hydroxyglutaric acid, and 3-hydroxycapric acid were produced in high amounts by *P. monteilii* L20, and these same compounds were significantly reduced from the supernatants by *P. nitroreducens* L16 (*P* < .05, Student’s *t* test) indicative of their importance for the cross-feeding interaction (Fig. 6E). The cross-feeding effects of 3-hydroxycapric acid on *P. nitroreducens* L16 were further experimentally confirmed using chemical standards (*P* < .001; Tukey's post hoc test) (Fig. 6E and S14). These results suggest that *P. nitroreducens* L16 and *P. monteilii* L20 formed a compositionally stable consortium with high K-solubilization efficiency due to commensal cross-feeding interaction.

## Discussion

**Figure 7:**
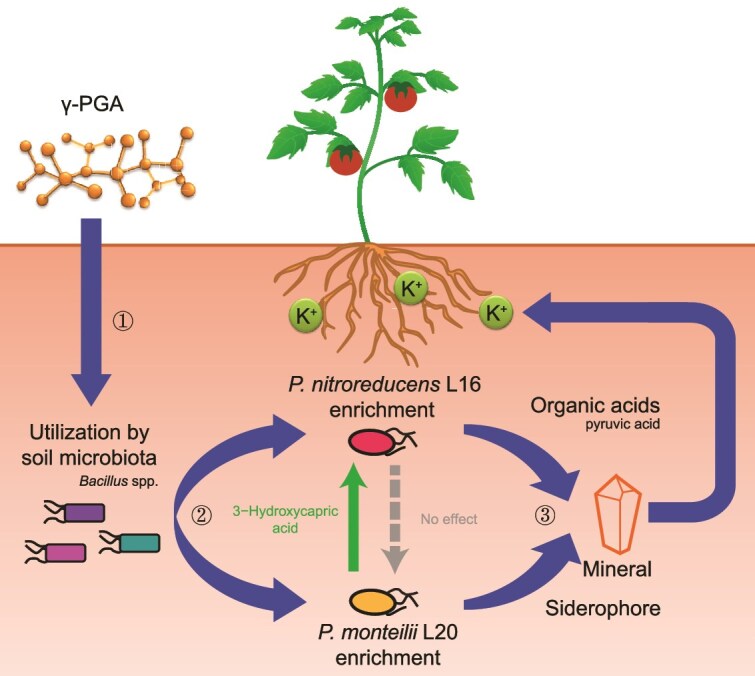
**Schematic drawing showing how γ-PGA application can drive the bacterial cross-feeding networks and plant growth-promotion.** The application of γ-PGA was utilized by soil microbiota, especially *Bacillus* spp., which further increased the abundance of *Pseudomonas nitroreducens* L16 and *Pseudomonas monteilii* L20 via cross-feeding (① and ②). The *P. nitroreducens* 16 and *P. monteilii* L20 also formed a stable cross-feeding consortium and solubilized K via secretion of organic acids and siderophores, leading to increased K content in tomato plants (③).

Manipulating microbiomes for improved plant productivity remains challenging and it is largely unclear how complex substrates that are microbially degraded to smaller breakdown products and metabolites influence the microbe-microbe interactions and plant growth-promotion via cross-feeding in rhizosphere microbiomes. In this study, we addressed whether microbial extracellular polymer γ-PGA can be applied to drive the rhizosphere microbial interactions for plant growth-promotion. We focused on N, P, and K as they are three major macronutrients needed for plant growth. We experimentally demonstrate that γ-PGA enhanced the biomass and K content of tomato by changing rhizosphere bacterial community assembly (Fig. 7). By using bulk soil for soil conditioning experiments, we could remove any potential γ-PGA-microbiome-plant-microbiome feedback, where inclusion of plants could have changed the soil biochemistry e.g. via root exudation in response to microbiomes. While this approach comes with a trade-off of losing direct effects of γ-PGA on plants in terms of N and P assimilation, we decided to focus on γ-PGA effects on microbiomes in soil conditioning experiment as microbiomes played the key role for increasing plant biomass and K content. However, studying the direct effects of γ-PGA on plant nutrient assimilation in the future is required to fully understand how these compounds drive the plant growth promotion. In addition to K and plant biomass, we found that γ-PGA increased the N and P contents of plants in the absence and presence of microbiota, the mechanisms underlying γ-PGA-mediated plant growth-promotion hence require more study in the future.

The γ-PGA increased the availability of K through enriching the abundance of K-solubilizing species such as *P. nitroreducens* L16 and *P. monteilii* L20. Mechanistically, these species solubilized K through two mechanisms: acidification (*P. nitroreducens* L16) and production of siderophores as K chelating agent (*P. monteilii* L20). As *P. nitroreducens* L16 secreted very similar amount of oxalic acid but higher amount of pyruvic acid than *P. monteilii* L20, pyruvic acid could be the effective compound responsible for K solubilization by *P. nitroreducens* L16. Although K-solubilizing bacteria frequently secrete siderophores and production of chelated compounds has been suggested to improve potassium availability [56], direct evidence of the role of siderophore in K-solubilization has been lacking. Here, we found that secretion of siderophores by *P. monteilii* L20 was the key mechanism in K-solubilization, as the K-solubilization was lost when the secreted siderophores were neutralized using specific culture medium. While more research is needed to determine which specific types of siderophores were involved in K-solubilization, the siderophores secreted by *P. monteilii* L20 could potentially chelate insoluble Al from K minerals, such as feldspar or potassium aluminum silicate [64, 65]. Additionally, the siderophores secreted by *P. monteilii* L20 could potentially have provided plants access to iron or other metals. Although another isolate zOTU_9 (most similar to *E. ludwigii* EN-119) was not significantly enriched by the γ-PGA-conditioned microbiome, it had also a strong K-solubilizing ability and could have affected K availability. While our results suggest that soil microbiome boosted plant growth via γ-PGA-induced K-solubilization, it is possible that organic acids and siderophores secreted by *P. nitroreducens* L16 and *P. monteilii* L20 could also have promoted the assimilation of other nutrients by tomato plants. As a result, the beneficial effects of γ-PGA on plant biomass could have been due to overall improvement of nutrient solubilization by microbiota, instead of K solubilization—a hypothesis that needs to be tested in the future.

Although we used 50% MS medium to supplement the potentially lost soil nutrients during autoclaving when preparing the microbial inocula for the transplant experiment, autoclaving likely affected the soil nutrient availability [66], which could have affected the nutrient availability to plants and the bacteria that were enriched in γ-PGA treatments. However, this effect was the same for both control and γ-PGA treatment and hence unlikely caused any systematic bias to our results. While we used four different types of agar plates to isolate rhizosphere microbes, certain microbes could not be successfully retrieved by these standard isolation procedures, and we were only able to obtain four of the 11 zOTUs of species that were enriched by γ-PGA-conditioned microbiota transplants. As a result, also other K-solubilizing bacteria that could not be isolated might have played role in γ-PGA-induced plant growth-promotion. In this study, species identification was based on 16S rRNA gene amplicon sequences and more information based on the whole genome sequences of the isolates is needed in the future for a more accurate species identification and characterization. Furthermore, we only focused on the role of bacterial community in γ-PGA-induced plant growth-promotion and the role of other important soil microbes, such as fungi and protists, need to be explored in future studies. The differences in the control plant K contents between different experiments in our study (Fig. 1A, 1D, 2C, and  3C) were likely due to the use of different culture systems (e.g. pot or tissue culture bottle system), variation in nutrient conditions (e.g. soil weight and application rate of MS medium), and differences in incubation time. For example, tomato plants of the first greenhouse experiment were collected when plants were 4 weeks old (Fig. 1A), while in other experiments plants were collect at 5 weeks old. Together, our results suggest that rhizobacteria can solubilize soil K for plants and may serve as a viable alternative to expensive and environmentally unfriendly K-fertilizers [67]. The effects of γ-PGA on the rhizosphere bacterial community assembly and plant K content provide new avenues for managing microbiome-driven plant growth-promotion.

Although the abundance of *P. nitroreducens* L16 and *P. monteilii* L20 were enriched by γ-PGA, neither of the two strains could utilize γ-PGA. Instead, resident microbiota, especially *Bacillus* species, could utilize γ-PGA for their own growth and further secreted metabolites that could promote the growth of *P. nitroreducens* L16 and *P. monteilii* L20. Although cross-feeding is common in bacterial communities [68], little research exists on cross-feeding interaction in relation to plant rhizosphere microbiomes. However, previous research has demonstrated that application of bio-organic fertilizer that contained *Bacillus amyloliquefaciens* W19 reduced the levels of the *Fusarium* wilt of banana by enriching soil *Pseudomonas* species [69]. Our results show that microbial cross-feeding interactions can be facilitated by application of complex polymers that lead to facilitative interactions between microbes and the plant. In the future, it would be important to directly show that γ-PGA effects are indeed caused by cross-feeding networks. This could be achieved e.g. by employing synthetic bacterial communities with known characteristics using the plant system developed in these experiments.

The γ-PGA is a complex substrate and too large to directly be imported through the cell envelope. As a result, degradative enzymes are needed to convert γ-PGA to transportable forms before taking up by the enzyme-producing microbes [20, 70]. One explanation for our result that most of the γ-PGA-utilizing isolates belonged to *Bacillus* genus could be that γ-PGA is often produced by *Bacillus* species [27, 28], and *Bacillus* species also frequently contain genes encoding a group of enzymes associated with γ-PGA degradation such as γ-D/L-glutamyl hydrolase, γ-glutamyltranspeptidase, peptidoglycan hydrolases [33, 71]. Although we found that the secretions of *Bacillus* strains promoted the growth of *P. nitroreducens* L16 and *P. monteilii* L20 after utilizing γ-PGA, the identification of specific growth-promoting metabolites is still needed. Previously, it has been shown that levulinic acid and valeric acid secreted by *B. velezensis* SQR9 could be utilized by *Pseudomonas stutzeri* XL272 strain [7], and the role of these compounds could be tested in the future. Additionally, further studies are needed to test if *Bacillus* spp. and *Pseudomonas* spp. benefit from the improved plant growth e.g. by selectively feeding on specific plant exudates, which has been shown to take place with *Arabidopsis* and *P. protegens* [72]. Such plant-microbe feedback could potentially stabilize microbial cross-feeding networks, especially when the cross-feeding interactions are not mutual. In this study, the effects of γ-PGA and bacterial metabolites on the growth of the isolated rhizobacteria were determined based on OD_600_. While this method can introduce possible biases to density estimates due to changes in cell shape or by microbe-derived extracellular polymeric substances, it was chosen due to high number of experimental treatments (n = 462), which made the use of other methods, such as colony counting, infeasible.

We also found that coculturing *P. nitroreducens* L16 and *P. monteilii* L20 increased their K-solubilizing efficiency. This could be potentially explained by synergism between K solubilizing mechanisms by *P. nitroreducens* L16 and *P. monteilii* L20: secretion of organic acids and production of siderophores. Two different mechanisms could simply lead to more efficient K solubilization or even division of labor where different species specialize in two different K solubilization mechanisms, resulting in higher per capita effects. The final ratios of *P. nitroreducens* L16 and *P. monteilii* L20 converged to 1:1 ratio in lab cocultures regardless of highly contrasting initial differences, indicating a compositionally stable consortium. The complex relationships between microbes can be positive (i.e. mutualism and commensalism), negative (i.e. competition and ammensalism), or asymmetric (i.e. predation and parasitism) [4, 5, 73, 74]. We found that *P. nitroreducens* L16 and *P. monteilii* L20 formed commensal interaction, where *P. nitroreducens* L16 gained fitness benefits in the presence of *P. monteilii* L20 by being able to feed on 3-hydroxycapric acid, whereas the metabolites produced by *P. nitroreducens* L16 did not have a significant effect on *P. monteilii* L20. When the ratio of *P. monteilii* L20 was higher in the initial inocula, it could likely produce more metabolites that *P. nitroreducens* L16 could cross-feed on, resulting in improved growth of *P. nitroreducens* L16. Hence, the benefits of cross-feeding could have potentially outweighed the low relative initial density of *P. nitroreducens* L16 in cocultures with *P. monteilii* L20, resulting in similar convergence in their final ratios. To support this, we found that the fold density increases of *P. nitroreducens* L16 measured after 12 h of inoculation increased with higher proportion of *P. monteilii* L20 present in the initial inoculum (Fig. S12C), likely explaining the convergence of species ratios despite the difference in initial inoculation ratios. The 3-hydroxycapric acid is a short chain fatty acid (SCFA) derivative secreted by bacteria [75]. Polysaccharide of *Cyclocarya paliurus* plant was shown to increase the content of 3-hydroxycapric by enriching SCFA-producing bacterial taxa in the rat gut [76]. However, the mechanism of action of 3-hydroxycapric acid on microbial growth needs to be tested experimentally in the future. Moreover, it is unclear which effects potentially benefitted the *P. monteilii* L20 as the coexistence of these strains was highly stable despite the one-sided effects. One explanation for this could be that *P. monteilii* L20 grew relatively faster than *P. nitroreducens* L16 (Fig. 6C-6D), resulting in convergence of final species ratios even when the abundance of *P. nitroreducens* L16 was relatively higher in the initial inocula. This could be potentially verified by coupling transcriptomics with metabolomics in co-cultures to better understand the complex chemical interactions between these species both in the absence and presence of *Bacillus* species. Moreover, although laboratory studies provide high accuracy in resolving the mechanisms underlying microbial species interactions, these species might interact differently and be affected by the plant secretions in the rhizosphere. Further mechanistic studies are thus needed to directly test and verify these hypotheses.

Overall, our findings suggest that complex polymers can trigger cascading of cross-feeding networks that improve the functioning of complex plant rhizosphere microbiomes. In the future, it would be interesting to test if γ-PGA can promote the growth of other plants in addition to tomato as the underlying mechanisms related to solubilization of key nutrients are relevant for the growth of most plants and as *Pseudomonas* and *Bacillu*s are common rhizosphere bacteria. Moreover, further studies are needed to study if naturally γ-PGA producing strains could be used as rhizosphere biostimulants or if pure γ-PGA is required for triggering the plant growth-promoting effects. Finally, more work is required to increase the γ-PGA yields during microbial fermentation to bring down the manufacturing costs. In addition to providing an important step towards understanding the importance of microbial cross-feeding networks in the rhizosphere, our findings highlight the potential of using complex polymers for rhizosphere microbiome manipulation to improve plant growth.

## Supplementary Material

Supplementary_Material_wraf040

External_Database_S1_wraf040

## Data Availability

The raw sequencing data have been deposited to the NCBI sequence read archive under the accession numbers PRJNA759398 and PRJNA1197718.
