# Supplementary material for: Plant growth-promotion triggered by extracellular polymer is associated with facilitation of bacterial cross-feeding networks of the rhizosphere
Source: ISME J. 2025 Mar 3;19(1):wraf040. doi: 10.1093/ismejo/wraf040 (PMC11937826; doi:10.1093/ismejo/wraf040)
Supplement: Supplementary_Material_wraf040 [file supplementary_material_wraf040.pdf]

Supplementary Material

This file includes:

Appendix S1 to S8

Fig. S1 to S14

Supplementary Materials and Methods

Appendix S1: Soil used in this study

The soil used in this study was collected from a tomato field in Pukou, Nanjing, China. Topsoil (0-20 cm) collected from the tomato field was air-dried, sieved (< 2 mm), and homogenized thoroughly. The soil in this area is yellow–brown earth (Udic Argosol) with pH of 6.8, available K of 133 mg kg^{-1}, available P of 41.5 mg kg^{-1}, NO_3^--N of 9.2 mg kg^{-1}, NH_4^+-N of 6.7 mg kg^{-1}, and organic matter content of 12.8 g kg^{-1}. We added vermiculite to the soil to protect tomato roots from mechanical injury when collecting rhizosphere soil at the end of the experiment.

Appendix S2: Microbiome profiling

To quantify γ-PGA effects on the microbiome composition, 0.3 g of rhizosphere soil samples were used for DNA extraction with Qiagen PowerSoil DNA extraction kit following the manufacturer's instructions. DNA concentrations were quantified using a NanoDrop spectrophotometer (ThermoScientific, WI, USA). For the 16S rRNA gene amplicon analysis, the V4-V5 region was amplified using the 515F/907R primers [1] with sample-specific barcodes following the PCR conditions as described previously [2]. Amplicons of 16S rRNA were sequenced on a Miseq instrument (Illumina, 2 × 250 bp) at Personal Biotechnology Co., Ltd. (Shanghai, China).

Raw sequencing data were processed using the UPARSE pipeline [3]. Briefly, pair of reads in FASTQ format of each sample was assembled, reads shorter than 370 bp were discarded, and low-quality nucleotides were filtered with a maximal expected error threshold of 1.0 to output a FASTA file using the commands fastq_mergepairs, fastx_truncate, and fastq_filter, respectively. After finding unique sequences, quality-filtered unique sequence reads were clustered into 100% sequence similar zero-radius operational taxonomic units (zOTUs) [4] using the command unoise3. zOTUs with fewer than eight reads were discarded, followed by filtration of chimeric reads. We obtained 65,294 ± 1,247 sequences per sample and all the samples were normalized to the depth of the smallest sample (53,506 reads). Taxonomic assignments of the zOTUs were obtained using the Ribosomal Database Project (RDP) pipeline (Taxonomy 18) [5] and NCBI.

Appendix S3: K solubilization by rhizobacteria

Solid Aleksandrov medium with K-feldspar powder as the sole source of K [6] was used to identify K-solubilizing bacteria. After 7 days of incubation, bacteria that showed solubilization zones on the agar plates were identified as potentially K-solubilizing bacteria. To quantitatively test the K-solubilization ability of these isolates, 1% bacterial suspension (OD_{600} = 0.5, ~10^7 CFU ml^{-1}) was inoculated to liquid Aleksandrov medium. After 9 days of incubation, the soluble K content in supernatants was determined using flame atomic absorption spectrometer (Varian Spectra AA 220 FS, Victoria, Australia) at 766.5 nm.

Appendix S4: Effects of *P. nitroreducens* L16 and *P. monteilii* L20 on plant growth-promotion

To determine the effects of *P. nitroreducens* L16 and *P. monteilii* L20 on plant growth-promotion, we inoculated overnight-grown bacterial suspension to pots (at a final concentration of 8×10^6 CFU g^{-1} soil), which contained 50 g of soil and one 7 day-old tomato seedling. Control treatments received an equal volume of sterile distilled water instead of bacterial inocula. Each treatment consisted of five replicates (n = 5) and each replicate contained three pots with one seedling. Each pot was fertilized with sterile MS liquid medium (15 mL) once per week and plants were collected at four weeks post inoculation. Plant biomass and K content were determined.

Appendix S5: Determining the siderophore production by *P. nitroreducens* L16 and *P. monteilii* L20

To determine the siderophore production by *P. nitroreducens* L16 and *P. monteilii* L20, we inoculated 5% bacterial suspension (OD_{600} = 0.5) to 100 mL iron-limited MKB medium (siderophore production is induced in iron-limited condition) [7] and MKB medium receiving 5% sterile distilled water was used as a control. Each treatment was replicated three times (n = 3). After 48 h of incubation, the siderophore content in liquid medium was determined using CAS assay [8] and siderophore production was compared between bacterial isolates using relative siderophore units as described previously [9]. Siderophore units were calculated as = [(absorbance of sample - absorbance of reference in blank medium)/absorbance of reference in blank medium]$\times$100% [10,11].

Appendix S6: Determining the effects of the metabolites secreted by γ-PGA-utilizing rhizobacteria on the growth of *P. nitroreducens* L16 and *P. monteilii* L20

To determine whether the metabolites of the γ-PGA-utilizing rhizobacteria can promote the growth of *P. nitroreducens* L16 and *P. monteilii* L20, we inoculated 1% suspension of the γ-PGA-utilizing rhizobacteria (OD_{600} = 2.5) to OS minimal medium supplemented with 1 g L^{-1} γ-PGA as the sole carbon source. After 36 h of incubation, the metabolites of each rhizobacteria were filtered to remove bacterial cells. The effects of these metabolites on the growth of *P. nitroreducens* L16 and *P. monteilii* L20 were studied on 96-well plates with each well containing 20 μL metabolites of each rhizobacteria after utilizing γ-PGA, 170 μL fresh OS minimal medium, and 10 μL of *P. nitroreducens* L16 and *P. monteilii* L20 bacterial suspensions (OD_{600} = 2.5). As a control, 96-well plates with each well containing 190 μL fresh OS minimal medium and 10 μL of *P. nitroreducens* L16 and *P. monteilii* L20 bacterial suspensions (OD_{600} = 2.5) were set. Each treatment was replicated three times (n = 3). After incubation for 24 h, the growth of *P. nitroreducens* L16 and *P. monteilii* L20 was determined based on $OD_{600.}$

Appendix S7: Testing the metabolic interactions between *P. nitroreducens* L16 and *P. monteilii* L20

To test metabolic interactions between *P. nitroreducens* L16 and *P. monteilii* L20, we grew them in the presence of each other's supernatants. Briefly, we inoculated 1% bacterial suspension of *P. nitroreducens* L16 or *P. monteilii* L20 (OD_{600} = 0.5) to LB medium. After 48 h of incubation, culture medium was filter-sterilized to obtain metabolites of *P. nitroreducens* L16 and *P. monteilii* L20. We then inoculated 2 μL bacterial suspension of *P. nitroreducens* L16 (OD_{600} = 2.5) to 200 μL mixture containing 20 μL metabolites of *P. monteilii* L20 and 180 μL fresh LB medium using 96-well plates. Similarly, we inoculated 2 μL bacterial suspension of *P. monteilii* L20 (OD_{600} = 2.5) to 200 μL mixture containing 20 μL metabolites of *P. nitroreducens* L16 and 180 μL fresh LB medium. 200 μL fresh LB medium which was inoculated with 2 μL bacterial suspension of *P. nitroreducens* L16 or *P. monteilii* L20 were set up as controls. Each treatment was replicated six times (n = 6) and cell growth was determined based on OD_{600}.

Appendix S8: UHPLC-MS/MS analysis

Separation was performed using a Vanquish UHPLC system (Thermo Fisher Scientific, USA) with a UPLC HSS T3 column (2.1 mm × 100 mm, 1.8 μm). The solvent system consisted of 5 mmol L^{-1} ammonium acetate and 5 mmol L^{-1} acetic acid in water (pH = 9.75) (eluent A) and acetonitrile (eluent B). A Q-Exactive HF-X mass spectrometer (Orbitrap MS, Thermo Fisher Scientific, USA) was used to determine MS/MS spectra on information-dependent acquisition (IDA) mode with spray voltage of 4.0 kV (positive) and -3.8 kV (negative), aux gas flow rate of 10 arb, sheath gas flow rate of 30 arb, and capillary temperature of 350 °C. Additive ions, molecular ion peaks, and fragment ions were used to predict the molecular formula. The raw data processing, peak detection, extraction, alignment, and integration were conducted by following the method described previously [12].

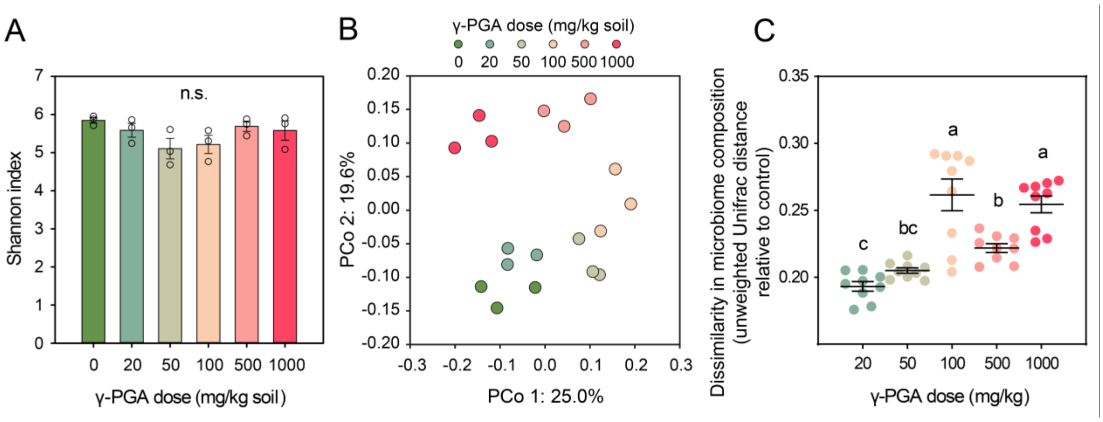

Fig. S1. Effects of γ-PGA concentrations on bacterial community alpha diversity (Shannon index; A) and beta diversity (Bray-Curtis distance; B) in the tomato rhizosphere and phylogenetic dissimilarity relative to control treatment (C). In panels A and C, error bars indicate means ± SE and small letters denote for statistically significant differences between different concentration treatments (ANOVA).

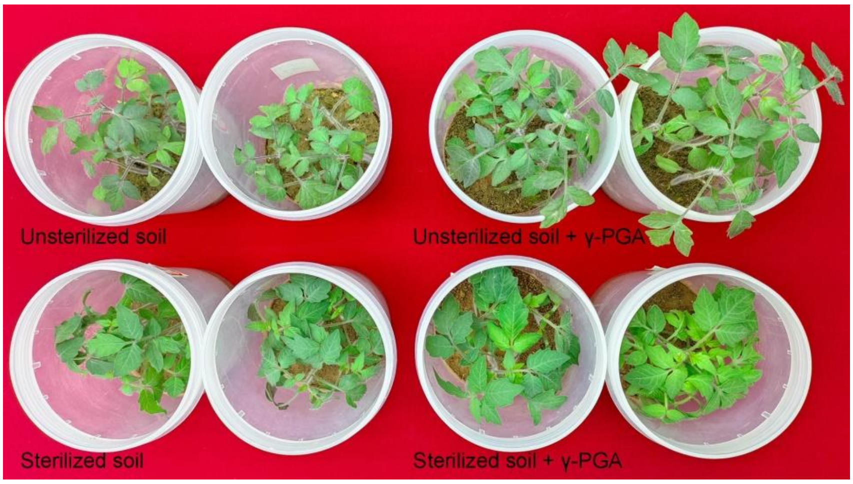

Fig. S2. The effect of γ-PGA (1000 mg kg^{-1} soil) on tomato plant growth in sterilized (bottom row) and unsterilized soil (microbiome present; top row) in the absence (left) and presence of γ-PGA (right).

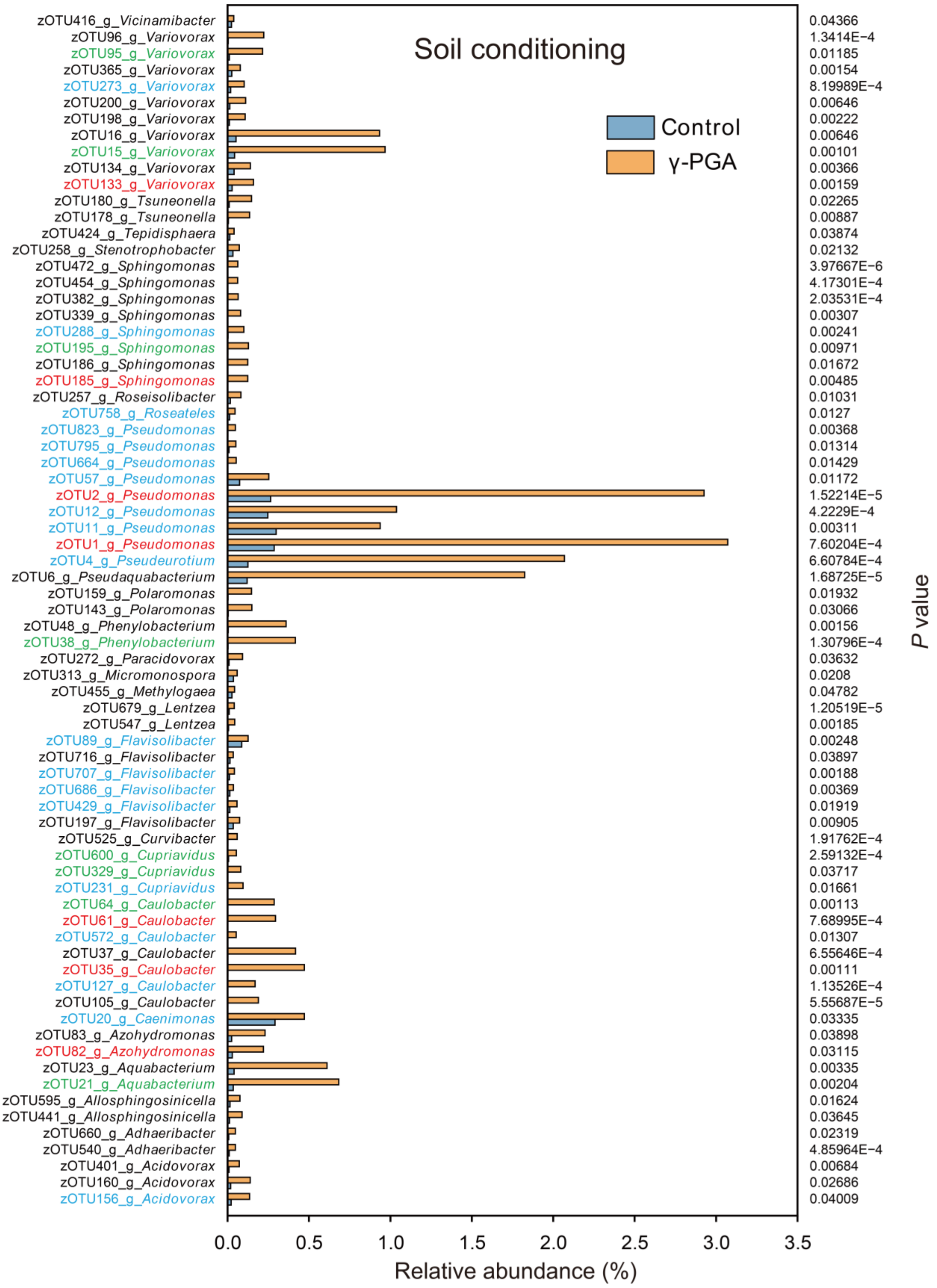

Fig. S3. Zero-radius zOTUs significantly enriched by soil conditioning with γ-PGA relative to water-conditioning in the control treatment (top 15% OTUs with highest average relative abundances in both control and γ-PGA conditioning treatments with *P* < 0.05). The average relative abundances of the top 15% zOTUs ranged between 0.2% - 1.7%

adding up to 59.0% of all sequences. *P*-values were calculated using Student's *t* test. Zero-radius OTUs that showed higher relative abundances in the rhizosphere by γ-PGA-conditioned microbiome transplants (Mapping threshold: score of 320, 97% similarity, and e value of $1e^{-150}$) are highlighted on blue ($P < 0.05$), green ($P < 0.01$), or red ($P < 0.001$) font.

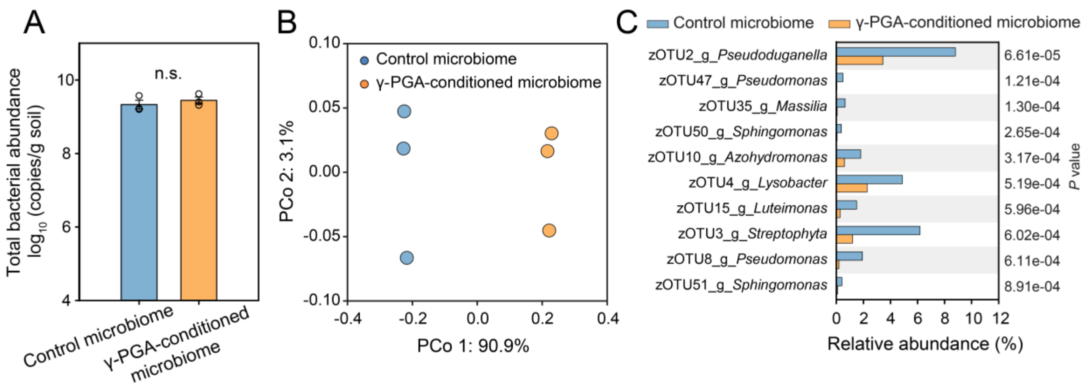

Fig. S4. Effects of control and γ-PGA-conditioned microbiota transplant on the total bacterial abundances (16S rRNA copy numbers per gram of soil; A) and the bacterial community beta diversity (Bray-Curtis distance; B) in the tomato rhizosphere at the end of the microbiota transplant experiment and top zOTUs (15%) significantly reduced (*P* < 0.001) in the rhizosphere of γ-PGA-conditioned microbiome treatment relative to control treatment at the end of the transplant experiment (C). In panel A, error bars indicate means ± SE.

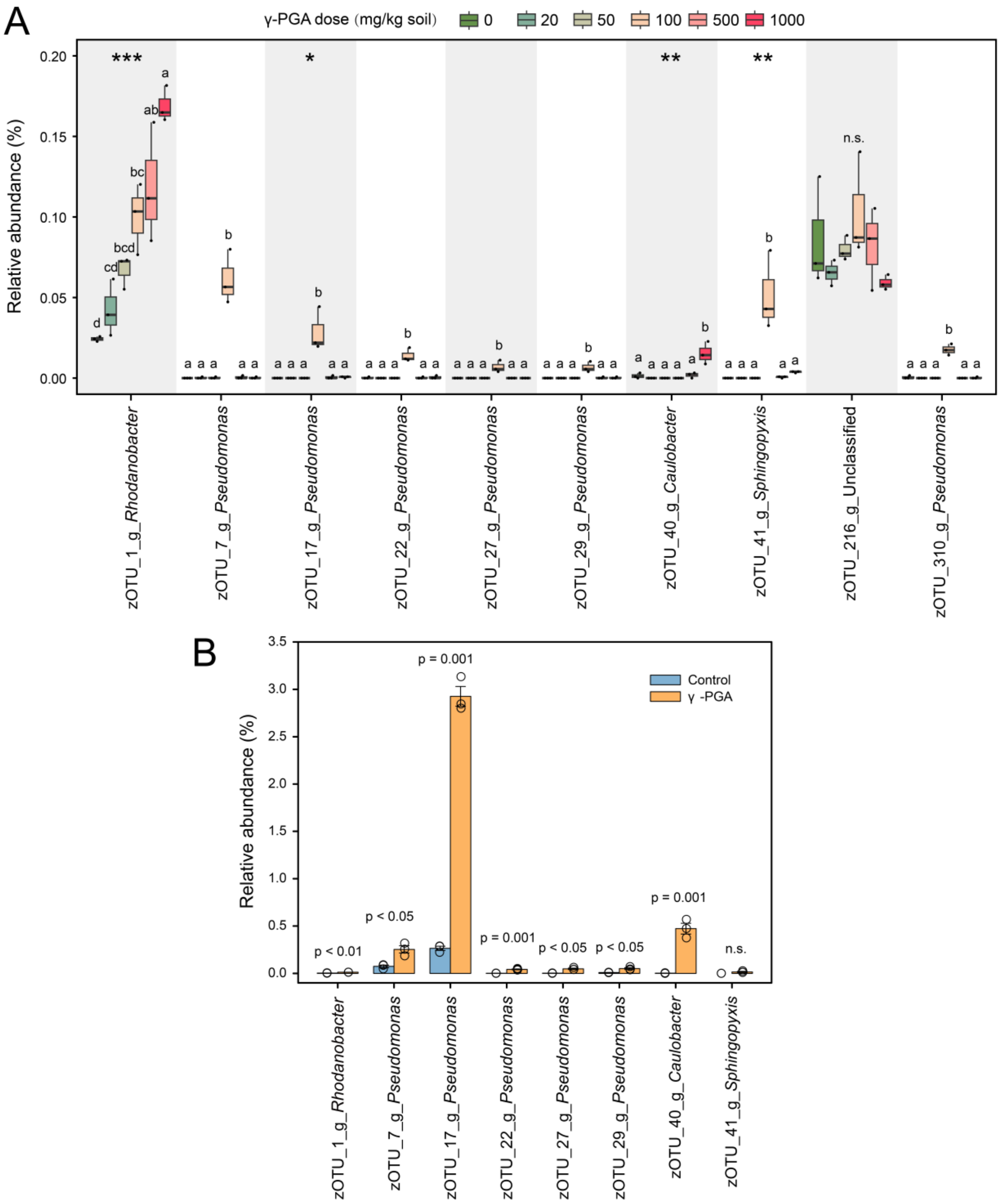

Fig. S5. The relative abundance of zOTUs in the first greenhouse experiment (A) and the soil conditioning experiment (B), which were significantly enriched in the rhizosphere by γ-PGA-conditioned microbiota transplant (as indicated in Fig. 2F). Mapping threshold: score of 320, 97% similarity, and e value of $1e^{-150}$. In panel A, small letters denote for statistically significant differences between different concentration treatments and asterisks indicate statistically significant correlations between the relative abundance of zOTUs and

γ-PGA dose (*** $P < 0.001$, ** $P < 0.01$, * $P < 0.05$; Spearman correlation). In panel B, error bars indicate means ± SE.

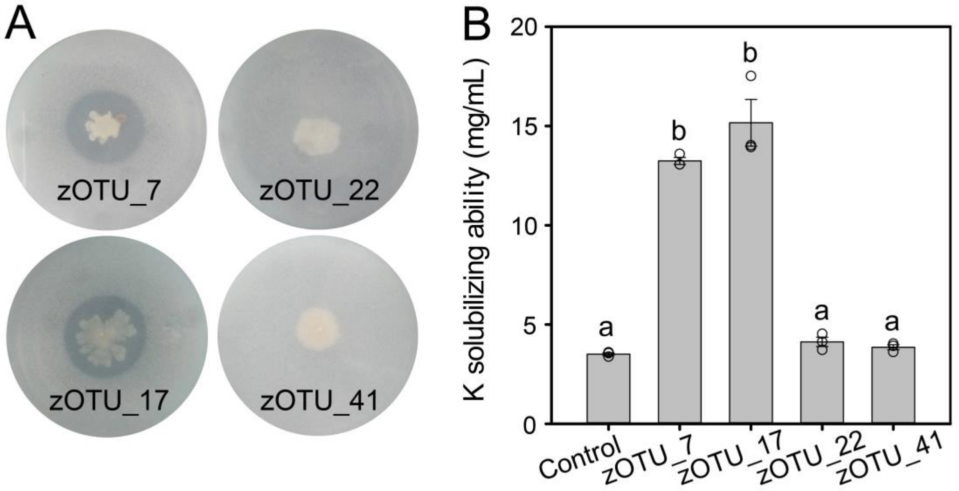

Fig. S6. Potassium-solubilizing ability of zOTU_7 (strain L20), zOTU_17 (strain L16), zOTU_22, and zOTU_41 in qualitative (A) and quantitative (B) tests. In panel B, error bars indicate means ± SE.

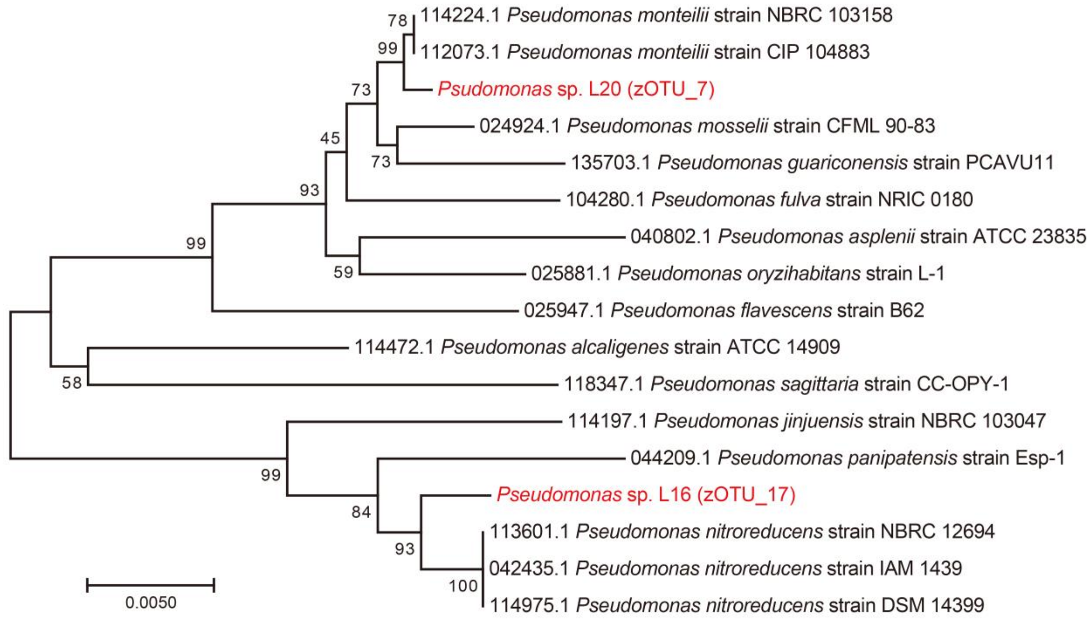

Fig. S7. Phylogenetic tree of zOTU_7 (strain L20) and zOTU_17 (strain L16) based on 16S rRNA gene sequences. Phylogenetic tree was constructed using the neighbor-joining method.

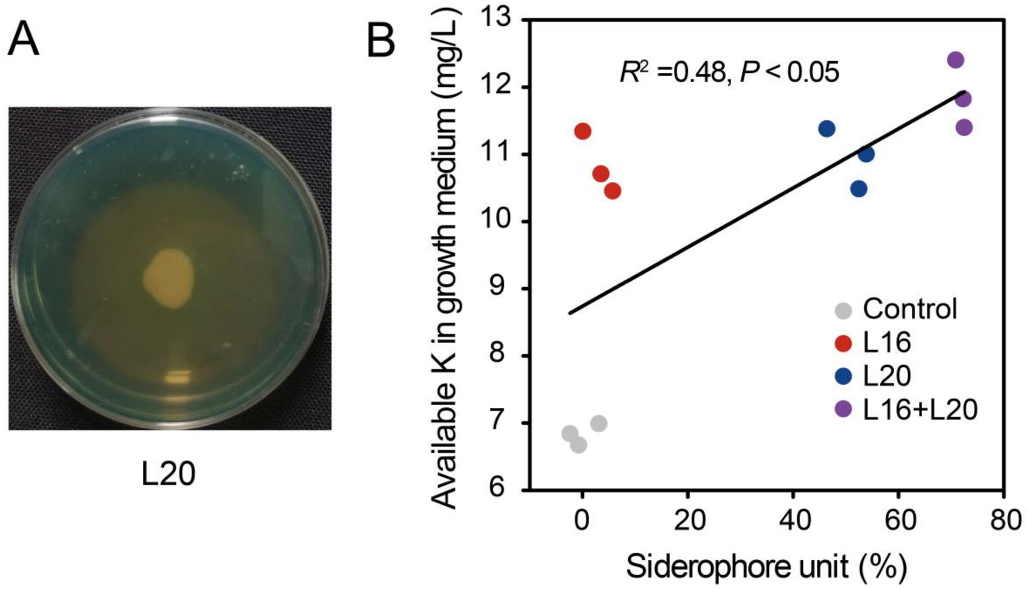

Fig. S8. ***P. monteilii*** **L20 likely solubilized potassium through production of siderophores.** (**A**) Siderophore producing zone (yellow zone) of *P. monteilii* L20 (zOTU_7) on CAS agar plate. (**B**) Correlation between the siderophore production ability of *P. nitroreducens* L16 and *P. monteilii* L20 in mono and co-cultures with available K content in liquid medium containing K minerals.

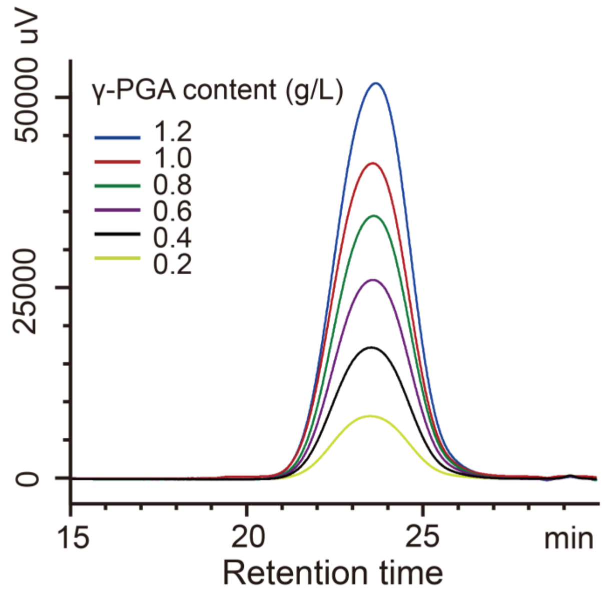

Fig. S9. HPLC chromatograms of different concentrations of γ-PGA.

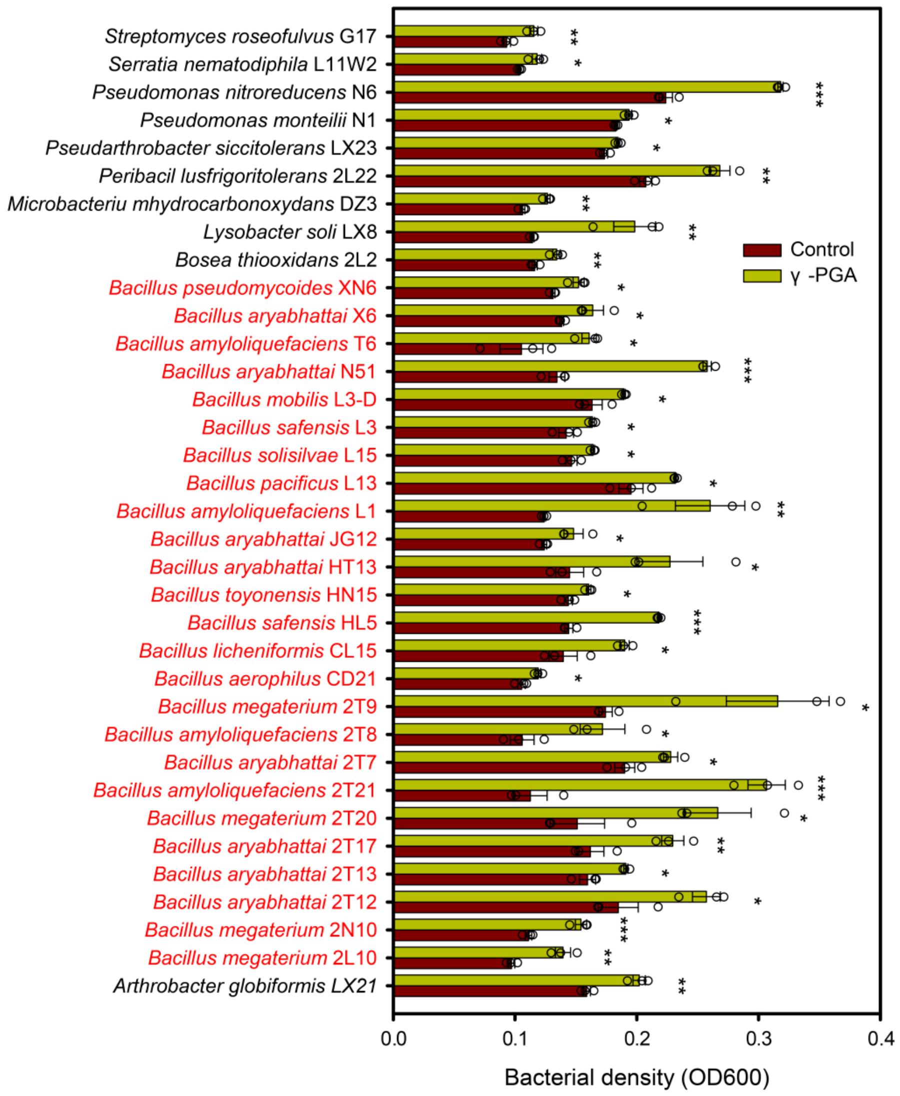

Fig. S10. Growth promotion of γ-PGA on the isolated rhizobacteria observed in microbiota transplant experiment. Rhizobacteria which were significantly promoted by γ-PGA are only shown (*, $P < 0.05$; **, $P < 0.01$; ***, $P < 0.001$). Isolates belonging to *Bacillus* spp. are highlighted on red font. Error bars indicate means ± SE.

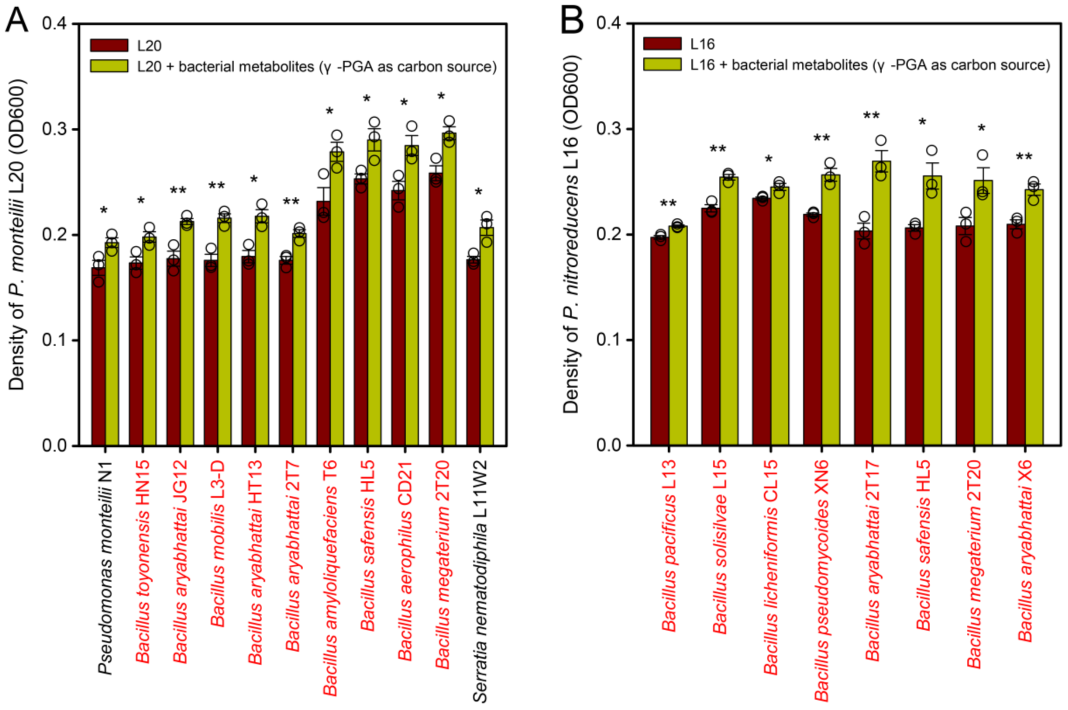

Fig. S11. Growth promotion of *P. monteilii* L20 (A) or *P. nitroreducens* L16 (B) by metabolites secreted by isolated rhizobacteria. This test was done by growing the isolates in minimal medium supplemented with γ-PGA as the sole carbon source and then extracting their supernatants to observe their effects on the growth of *P. nitroreducens* L16 and *P. monteilii* L20. Isolates that secrete metabolites, which significantly promote the growth of *P. monteilii* L20 or *P. nitroreducens* L16 are only shown (*,$P < 0.05$; **, $P < 0.01$; ***, $P <$ 0.001). Isolates belonging to *Bacillus* spp. are highlighted on red font. Independent control for each bacterial metabolite was included to correct for batch effects and edge effects of culturing *P. monteilii* L20 and *P. nitroreducens* L16 in 96-well plates. In all panels, error bars indicate means ± SE.

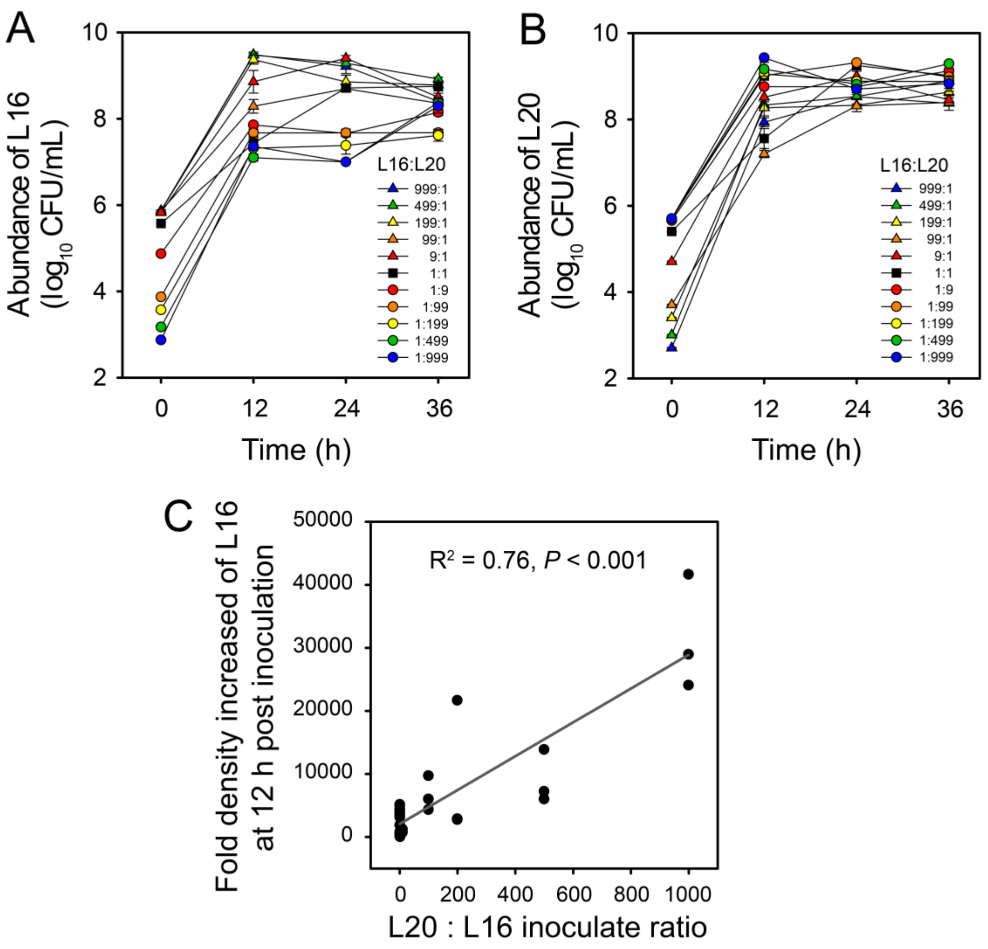

Fig. S12. Temporal dynamics in *P. nitroreducens* L16 (A) and *P. monteilii* L20 abundances (B) during coculturing with different inoculation ratios and the correlation between fold density increase of *P. nitroreducens* L16 after 12 h of inoculation across increasing inoculation ratios of *P. monteilii* L20 (C). The fold density increase of L16 = (abundance of L16 at 12 h - initial abundance of L16) / initial abundance of L16. In panels A and B, error bars indicate means ± SE.

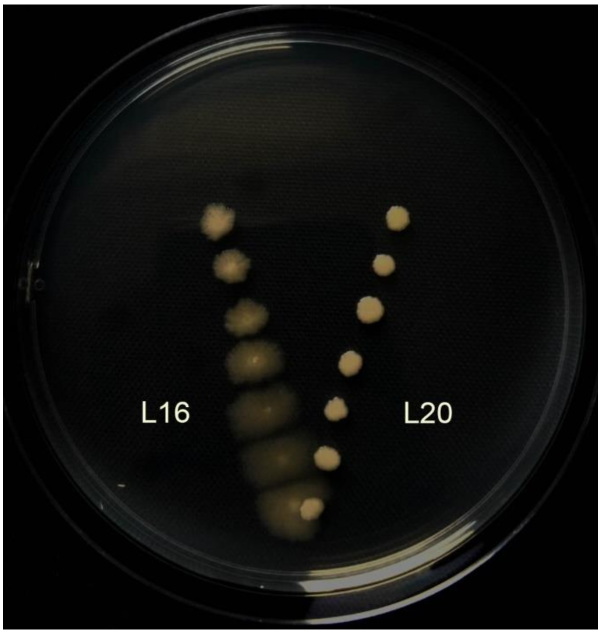

Fig. S13. Attraction between colonies of *P. monteilii* L20 and *P. nitroreducens* L16 on LB agar plate. The colony diameters of *P. nitroreducens* L16 were increased in the vicinity of *P. monteilii* L20 indicative of attraction.

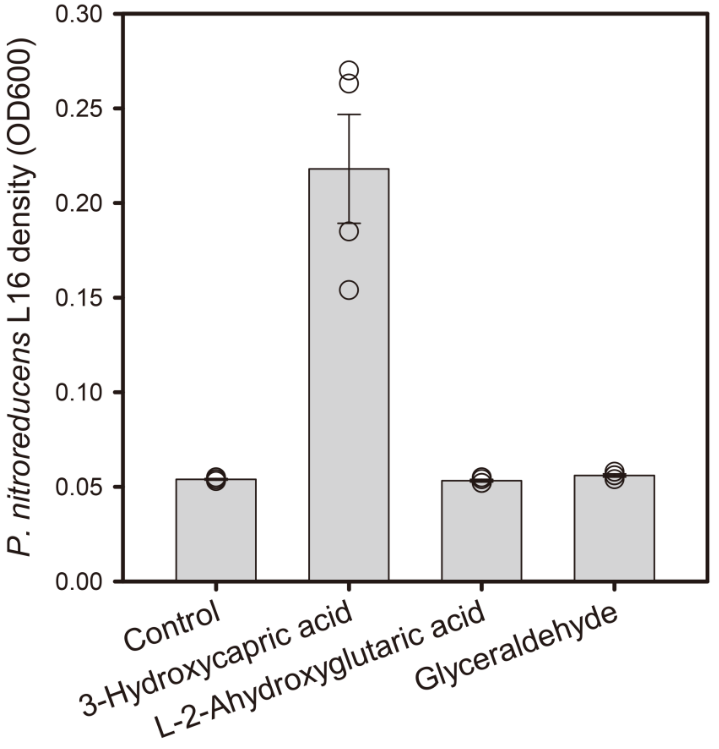

Fig. S14. Experimental verification of the effects of metabolites secreted by *P. monteilii* L20 on the growth of *P. nitroreducens* L16. Error bars indicate means ± SE.

Reference

1. Biddle JF, Fitz-Gibbon S, Schuster SC, Brenchley JE, et al. Metagenomic signatures of the Peru Margin subseafloor biosphere show a genetically distinct environment. Proc Natl Acad Sci USA. 2008;**105**: 10583-10588.
2. Gu Y, Wang X, Yang T, Friman VP, et al. Chemical structure predicts the effect of plant-derived low molecular weight compounds on soil microbiome structure and pathogen suppression. Funct Ecol. 2020;**34**: 2158-2169.
3. Edgar RC. UPARSE: highly accurate OTU sequences from microbial amplicon reads. Nat Methods. 2013;**10**: 996-998.
4. Edgar RC. UNOISE2: improved error-correction for Illumina 16S and ITS amplicon sequencing. BioRxiv. 2016: 081257.
5. Wang Q, Garrity GM, Tiedje JM, Cole JR. Naive Bayesian classifier for rapid assignment of rRNA sequences into the new bacterial taxonomy. Appl Environ Microbiol. 2007;**73**: 5261-5267.
6. Zhang C, Kong F. Isolation and identification of potassium-solubilizing bacteria from tobacco rhizospheric soil and their effect on tobacco plants. Appl Soil Ecol. 2014;**82**: 18-25.
7. Seong K-Y, Höfte M, Boelens J, Verstraete W. Growth, survival, and root colonization of plant growth beneficial *Pseudomonas fluorescens* ANP15 and *Pseudomonas aeruginosa* 7NSK2 at

different temperatures. Soil Biol Biochem. 1991;**23**: 423-428.
8. Schwyn B, Neilands J. Universal chemical assay for the detection and determination of siderophores. Anal Biochem. 1987;**160**: 47-56.
9. Gu S, Wei Z, Shao Z, Friman V-P, et al. Competition for iron drives phytopathogen control by natural rhizosphere microbiomes. Nat Microbiol. 2020;**5**: 1002-1010.
10. Ghosh SK, Bera T, Chakrabarty AM. Microbial siderophore – A boon to agricultural sciences. Biol Control. 2020;**144**: 104214.
11. Machuca A, Milagres AM. Use of CAS-agar plate modified to study the effect of different variables on the siderophore production by *Aspergillus*. Lett Appl Microbiol. 2003;**36**: 177-181.
12. Sun X, Xu Z, Xie J, Hesselberg-Thomsen V, et al. *Bacillus velezensis* stimulates resident rhizosphere *Pseudomonas stutzeri* for plant health through metabolic interactions. ISME J. 2021;**16**: 774-787.
